# Evolution of human H3N2 influenza virus receptor specificity has substantially expanded the receptor-binding domain site

**DOI:** 10.1016/j.chom.2024.01.003

**Published:** 2024-02-01

**Authors:** Andrew J. Thompson, Nicholas C. Wu, Angeles Canales, Chika Kikuchi, Xueyong Zhu, Beatriz Fernandez de Toro, Francisco J. Cañada, Charli Worth, Shengyang Wang, Ryan McBride, Wenjie Peng, Corwin M. Nycholat, Jesus Jimenez-Barbero, Ian A. Wilson, James C. Paulson

**Affiliations:** 1. Department of Molecular Medicine, The Scripps Research Institute, La Jolla, California, 92037, USA.; 2. Department of Integrative Structural and Computational Biology, The Scripps Research Institute, La Jolla, California, 92037, USA.; 3. Department of Organic Chemistry, Faculty of Chemistry, Universidad Complutense de Madrid, Avd. Complutense s/n, 28040, Madrid, Spain..; 4. Structural and Chemical Biology Department, Centro de Investigaciones Biológicas Margarita Salas, C/Ramiro de Maeztu 9, 28040 Madrid, Spain.; 5. CIBERES, ISCIII, 28029 Madrid, Spain; 6. CIC bioGUNE Bizkaia Science and Technology Park, 48160 Bilbao, Spain.; 7. IKERBASQUE, Basque Foundation for Science 48009 Bilbao, Spain.; 8. Department of Immunology & Microbiology, The Scripps Research Institute, La Jolla, California, 92037, USA.

## Abstract

Hemagglutinins (HAs) from human influenza viruses descend from avian progenitors that bind α2–3-linked sialosides, and must adapt to glycans with α2–6-linked sialic acids on human airway cells to transmit within the human population. Since their introduction during the 1968 pandemic, H3N2 viruses have evolved over the past five decades to preferentially recognize α2–6-sialoside receptors that are elongated through addition of poly-LacNAc. Using STD-NMR, X-ray crystallography, and solid-phase glycan microarrays, we show that more recent H3N2 viruses now make increasingly complex interactions with elongated receptors, while continuously selecting for strains maintaining this phenotype. This change is accompanied by an extension of the traditional receptor binding site to include residues in key antigenic sites on the surface of HA trimers. These results help explain the propensity for selection of antigenic variants, leading to vaccine mismatching, when H3N2 viruses are propagated in chicken eggs or cells that do not contain such receptors.

## Introduction:

Historically, and indeed until very recently, influenza A viruses (IAVs) have comprised the major infectious agent leading to severe respiratory disease and death among humans.^1^ For at least the last century, the annual, seasonal occurrence of relatively benign IAVs have been interspersed with more severe IAV epidemics and coupled with occasional global pandemics from which these seasonal viruses emerge. IAVs have proved debilitating to devastating in terms of human health, with cumulative estimated morbidities and mortalities from IAV-derived illness numbering from the hundreds of thousands to tens to hundreds of millions.^2,3^ All known human and many animal IAVs originate from a much larger natural reservoir of avian influenza viruses. Occasionally, avian IAV strains with enhanced potential to infect humans, typically derived from genetic reassortment between human, avian and swine viruses, are able to cross the species barrier (zoonosis) and transmit within the human population, leading to sporadic pandemics and subsequent emergence of novel endemic human IAV strains.^3–5^ Four human IAV strains (H1N1, H2N2, H3N2, and a subsequent distinct H1N1) have arisen in this way over the last approximately 100 years^3,6^. While other avian viruses periodically cause human infection (e.g. H5N1^7,8^, H7N9^9^), these zoonotic viruses have fortunately not been able to transmit from human-to-human^10–13^.

IAV strains are named according to the antigenic similarity or serotype of two major viral surface glycoproteins, hemagglutinin (HA or H) and neuraminidase (NA or N), which also play key roles in binding and release, respectively, from receptors on airway epithelial cells. Influenza HAs bind sialic acid (N-Acetylneuraminic acid, NeuAc) containing glycans (sialoglycans) on cell surface glycoproteins and glycolipids as their primary biological receptors.^10,11,14^ However, depending on their chemical configuration, these glycans can also act as a species-defined barrier to viral transmission. HAs from human IAVs exhibit “human-type” receptor specificity and bind selectively to glycans on cells lining the human airway which predominantly feature terminal sialic acids attached in the α2–6 configuration to galactose (NeuAcα2–6Gal), whereas HAs from avian viruses exhibit specificity for “avian-type” α2–3-linked (NeuAcα2–3Gal) receptors.^10,11,14–17^ Thus, for zoonotic avian viruses to be sustained within the human population, they must achieve substantial community spread, transmitting readily via respiratory droplets from person to person, in a process now widely shown to require, among other aspects, adaptation in the avian HA to bind human α2–6-linked sialoglycans.^1^

Within human pandemic IAV strains (H1, H2, and H3) of the early- to mid-20^th^ century, such changes were achieved via relatively simple two-amino acid substitutions leading to near-complete receptor specificity switches.^3,15,18–21^ However, it is now widely accepted that such early forms of these respective viruses represent only minimally adapted strains and, that although diagnostic, binding to α2–6 versus α2–3 sialosides that are synthetic terminal fragments of much larger natural glycan chains belies the true complexity of the underlying respiratory glycome, particularly within humans.^22–24^ Previously, we and others have demonstrated that over time, IAV HAs within the human H3 subtype have evolved more complex receptor specificity, selecting for elongated α2–6 sialoglycans featuring at least three linear LacNAc (galactose linked β1–4 to N-acetylglucosamine, Galβ1–4GlcNAc) repeats beneath the terminal species-defining sialoside moiety.^24–27^ Here we show that human H3N2 strains have largely maintained specificity for extended glycan receptors. By utilizing a variety of structural techniques, we provide evidence that this enhanced mode of receptor-binding has evolved over many years to include mutations outside the traditionally defined sialic acid receptor-binding site (RBS). Indeed, binding of extended glycan receptors now includes residues in the HA head domain that are typically under immune pressure, comprising antibody binding epitopes largely within antigenic site B in group 2 influenza viruses.^28^ We suggest that mutations arising from antigenic selection are further linked to receptor binding, such that immune selective pressure on H3N2 viruses results in variants that avoid neutralization but must also retain binding to human-type receptors to maintain fitness for transmission. In this regard, we also highlight a recent example where antigenic shift of H3 due to a new glycan within HA antigenic site B near the receptor binding site also required compensating mutations to maintain favorable binding to extended receptors. Our focus on human H3 HAs within this work offers fascinating insight into IAV biology since H3N2 viruses epitomize the ability of IAVs to continuously adapt, resulting in continuous circulation in the human population for over 50 years. Similarly to long-lived H1N1 strains emerging in 1918 and 1977 that each circulated for over 30 years, these viruses highlight the evolutionary path that novel strains can take on the transition from fast-moving, yet comparatively short-lived, pandemics to sustained seasonal endemic spread by continuous adaptation to avoid immune suppression yet maintain fitness for binding to host receptors.

## Results & Discussion:

### H3 hemagglutinins from dominant contemporary H3N2 viruses continue to bind preferentially to extended glycan receptors:

**Figure 1.**
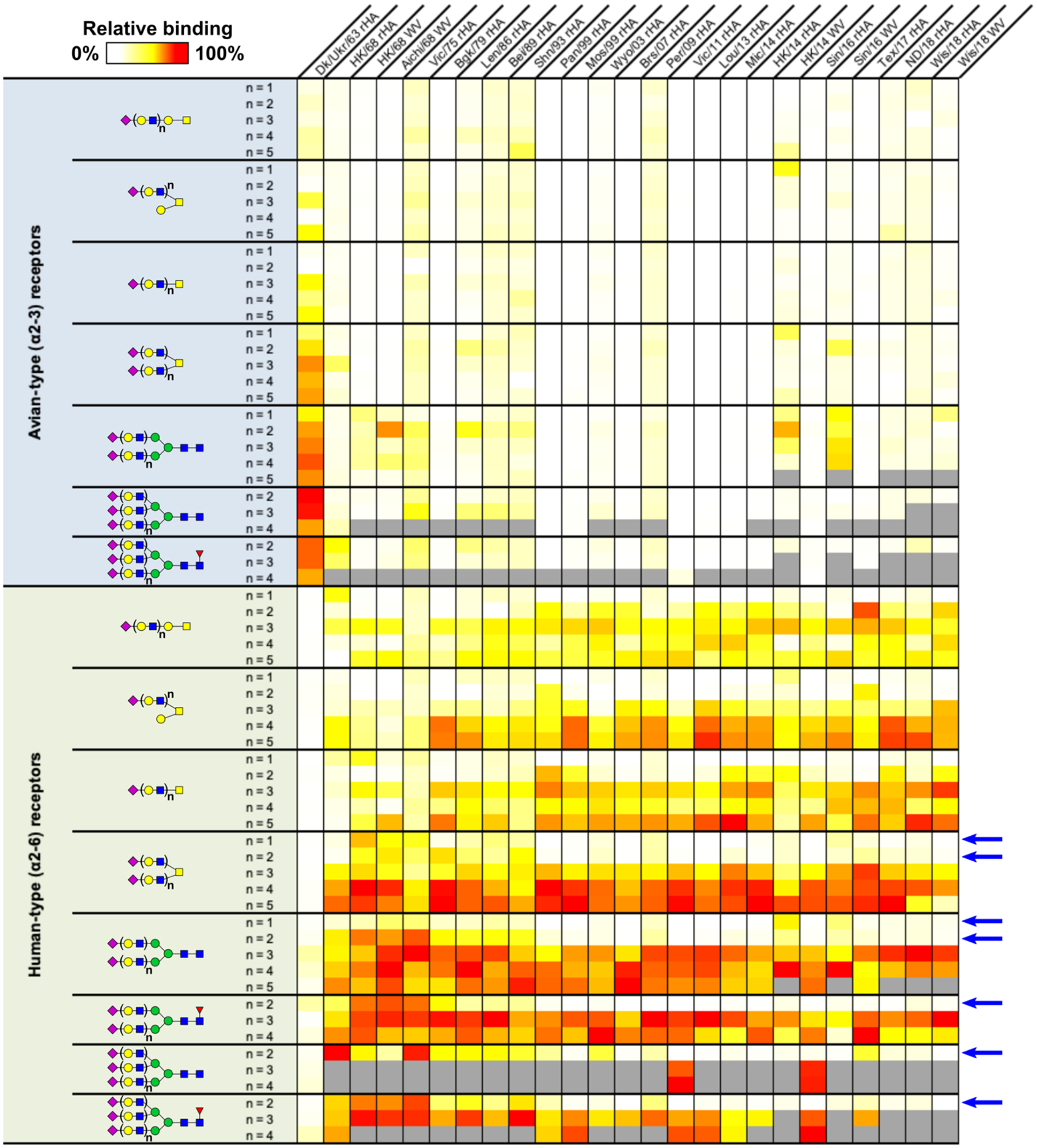
Glycan microarray analysis of WT H3 receptor specificity from pre-1968 pandemic to 2018. Heatmap representation of glycan microarray data comparing receptor binding specificity of major H3(N2) strains, including vaccine strains, over time. Viral strains are listed in abbreviated form across the top row and denoted as either recombinant hemagglutinin (rHA) or whole-virus (WV) samples. Over time, binding of human strains to shorter glycans containing only one or two LacNAc repeats beneath terminal sialic acid (highlighted via blue arrows to the right of the panel) are reduced and is eventually eliminated. The color scale within individual columns (array datasets) are independently scaled to the most intense RFU within that group. Columns depict data collected from various different Sialoside array versions, including V1, V3, V4, & V5. Receptor structures corresponding to glycan numbers for all array versions can be found in Supplementary Data S1, glycan diagrams shown are according to the Symbol Nomenclature for Glycans recommended by the NLM.^70,71^ Grey bars illustrate that a given receptor structure was absent in the array version on which a particular dataset was collected (see Supplementary Data S1). Individual array plots for all HAs are illustrated in Supplementary Data S2.

**Figure 2.**
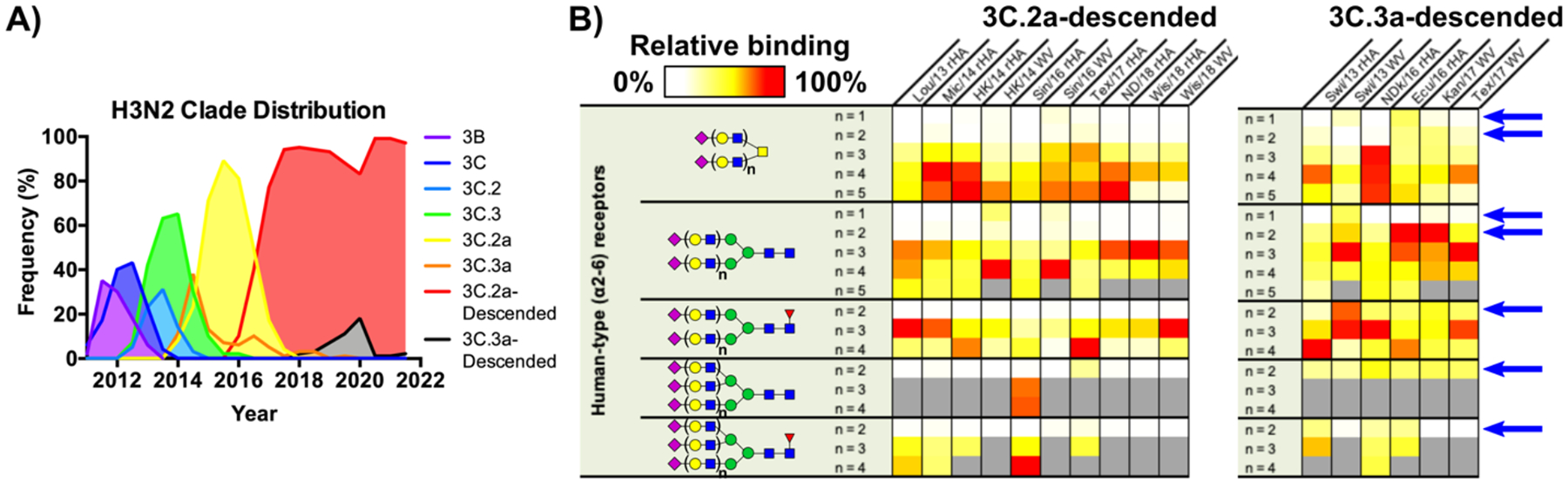
Clade distribution and receptor specificity of clades 3C.2a and 3C.3a (A) Frequency distribution of human H3N2 clades over the past decade (adapted from Nextstrain data) shows that individual clades arise and recede regularly. However, a major divergence in H3N2 phylogeny in approximately 2012 (see Fig. S2) subsequently led to clades descended from 3C.2a becoming dominant in recent years. (B) Heatmap representation of glycan microarray data (α2–6 sialosides only) for clades 3C.2a- and 3C.3a-descended strains. As shown, 3C.2a maintains length selectivity of prior dominant strains and are ultimately selected for, while 3C.3a viruses regain binding to short receptors (highlighted via blue arrows) and become outcompeted. The color scale within individual columns (array datasets) are independently scaled to the most intense RFU within that group. Columns depict data collected from various different Sialoside array versions, including V1, V3, V4, & V5. Receptor structures corresponding to glycan numbers for all array versions can be found in Supplementary Data S1. Grey bars illustrate that a given receptor structure was absent in the array version on which a particular dataset was collected (see Supplementary Data S1). Individual array plots for all HAs are illustrated in Supplementary Data S2.

In recent years, expanded glycan microarrays comprising tens to hundreds of intact N- & O-linked glycans and glycolipids, as well as key terminal fragments, have proved exceptionally valuable resources in dissecting IAV receptor binding interactions and the effects of mutations within the viral HA and NA surface proteins.^25,26,29–31^ Previously, employing a sialoside microarray comprising dozens of N-, O-linked, and lipid scaffolds and featuring LacNAc extensions varying between one and five repeats proximal to the terminal α2–3 and α2–6 sialic acid of sialosides, we reported detailed receptor-binding specificities for a panel of H3N2 viruses isolated between from their initial emergence in 1968 through to 2011 vaccine strains^26^. A major finding from this work was that, during the early 2000s, human H3N2 IAVs exhibited a narrowing of their previously broad α2–6 human-type receptor specificity to bind selectively to only extended versions of these glycans featuring linear chains of at least 3 LacNAc repeats underlying sialic acid (see Fig. S1). To characterize molecular determinants contributing to this phenotype and isolate specific amino acid variants driving the evolution of receptor specificity, we now present a greatly expanded sialoside microarray analysis featuring many additional H3N2 strains from 1968 – 2021, with particular emphasis on those emerging within the last decade (Fig. 1 & 2).

Employing a heatmap representation for ease of comparison, we analysed binding of recombinant hemagglutinins and whole viruses from 30 H3N2 IAVs to native N-linked and O-linked glycans that vary by length (Fig. 1). These strains include well-studied isolates over the last 50 years and recent vaccine strains from dominant clades that represent authentic viruses circulating in the human population. As revealed previously, the HAs from all H3N2 human IAVs generally show increasingly strong binding to sialoside receptors as a function of length.^10,26^ However, pandemic and early seasonal H3N2 strains from 1968 to approximately the mid 1970s clearly show little length selectivity, with observable binding to most receptors within a given structural group regardless of length (Fig. 1; see strains HK/68 to Vic/75). Interestingly, from the late 1970s through to the early 1990s (strains Bgk/79 to Shn/93), a marked decreased in binding to N-linked and biantennary O-glycans containing only one or two LacNAc repeats beneath the terminal sialoside is evident. In the late 1990s to early 2000s and onwards, this trend progresses, culminating in a receptor specificity phenotype that almost entirely eliminates HA-mediated binding to these shorter receptors. As highlighted in the right-hand columns of Figure 1, this restricted receptor specificity has persisted right through to the most recent strains available for analysis.^25–27,32^ Concurrently, the substantial narrowing of glycans available to act as receptors has presented challenges to IAV research, both in the lab and for vaccine development, since contemporary strains often fail to hemagglutinate conventional animal erythrocytes,^33–36^ and undergo HA and/or NA mutations that alter key receptor-binding or antigenic properties when grown in eggs or commonly used cell lines. Rapid appearance of such variants in culture are almost certainly due to mismatches in both length and terminal sialoside linkages between preferred receptor targets and those present on standard laboratory hosts.^10,37–40^

### Specificity for extended glycan receptors correlates with enhanced fitness in human hosts

Of particular interest is whether preferential binding to extended sialoside receptors, or alternatively loss of binding to shorter glycans, reflects an evolutionary adaptation conferring a selective advantage. With this in mind, we sought to further investigate receptor specificity of viral strains belonging to subdominant clades that have emerged in the recent past, but ultimately proved to be selected against, and whose circulation in humans has dropped to near-zero in favor of now-dominant alternatives. A prime example of this type of competitive selection within human H3N2 IAVs occurred during the early part of the last decade when clade 3C strains became dominant over a prior 3B branch (see Fig. 2A) and subdivided into two major lineages, clades 3C.2 and 3C.3, which cocirculated through 2013 – 2014 until their evolution into clades 3C.**2a** and 3C.**3a**, respectively. Subsequent strains and subclades evolved from these two major parental groups now represent nearly all H3N2 IAVs circulating in the human population to-date (see Fig. S4A).

Population-level virus sequencing and other epidemiological data have now shown that, since their emergence, clade 3C.**2a** and its descendants have rapidly established and largely maintained dominance as the major H3N2 seasonal strains throughout the last decade (see Fig. 2A & S4A).^41,42^ Notably, these dominant 3C.**2a**-descended viruses, harboring superior fitness traits within humans, have almost exclusively maintained the strict receptor-binding length selectivity phenotype observed in prior strains (see all post-2013 strains in Fig. 1). We thus sought to examine the receptor specificity of selected 3C.**3a**-descended strains to determine if altered preferences in receptor binding phenotype might be among those traits contributing to the ultimate selection against these viruses (Fig. 2A). Surprisingly, considering their extremely high sequence similarity to contemporary clade 3C.**2a** counterparts (Supplementary Data S3), a panel of six 3C.**3a** recombinant HAs and whole virus samples from isolates spanning five years from 2013 through 2017 all showed minimal selectivity for extended human-type sialoside receptors present on glycan microarrays, with widespread recovery of binding to glycans containing n=2 LacNAc repeats (Fig. 2B). The fact that human H3N2 IAVs have now twice (through both the 1990s and early 2010s) undergone competitive selection to maintain specific binding to extended receptors strongly highlights this phenotype not only as a key determinant of fitness, but also strongly suggest such glycan structures as important host receptors. Indeed, the observation of such rapid elimination of clade 3C.**3a** viruses from the human population, given that strains within this group descend from progenitors that all previously exhibited strong length-selective binding (i.e. clade 3C strains, including well-studied examples such as Vic/11 (A/Victoria/361/2011)), appears particularly poignant in this context.

### The molecular basis of length-selective receptor binding in human H3N2 influenza viruses

Determination of the precise mutations that contribute to switching from “avian-type” specificity (α2–3) to “human-type” specificity (α2–6) upon adaptation of avian viruses to humans are regarded as key in monitoring of high-risk variants in naturally circulating strains to provide early warning of avian IAVs with enhanced pandemic potential.^43–45^ For this reason, we have a longstanding interest to further identify the key HA residues where mutations have contributed to the evolution of length-selective receptor binding since this phenotype has been maintained over several decades and have dominated key H3N2 clades in humans. We anticipate that such data could usefully inform vaccine selection.

Since the early post-pandemic viruses, which bound comparably to both short and long sialoside receptors, there have been >20 conserved mutations proximal to and within the RBS in HA1. Many of these mutations are the result of antigenic drift, with some unrelated or only indirectly related to receptor binding. Thus, it is a challenge to identify and characterize individual or even combinations of variants underlying length selectivity. However, the observation that 3C.**3a**-descended strains, which bind both short and extended receptors, possess only very few mutations relative to clade 3C.**2a** contemporary viruses or 3C.3 progenitors (which maintain selection for elongated α2–6 sialoside structures) has provided an opportunity to gain valuable insights into receptor-length preferences.

**Figure 3.**
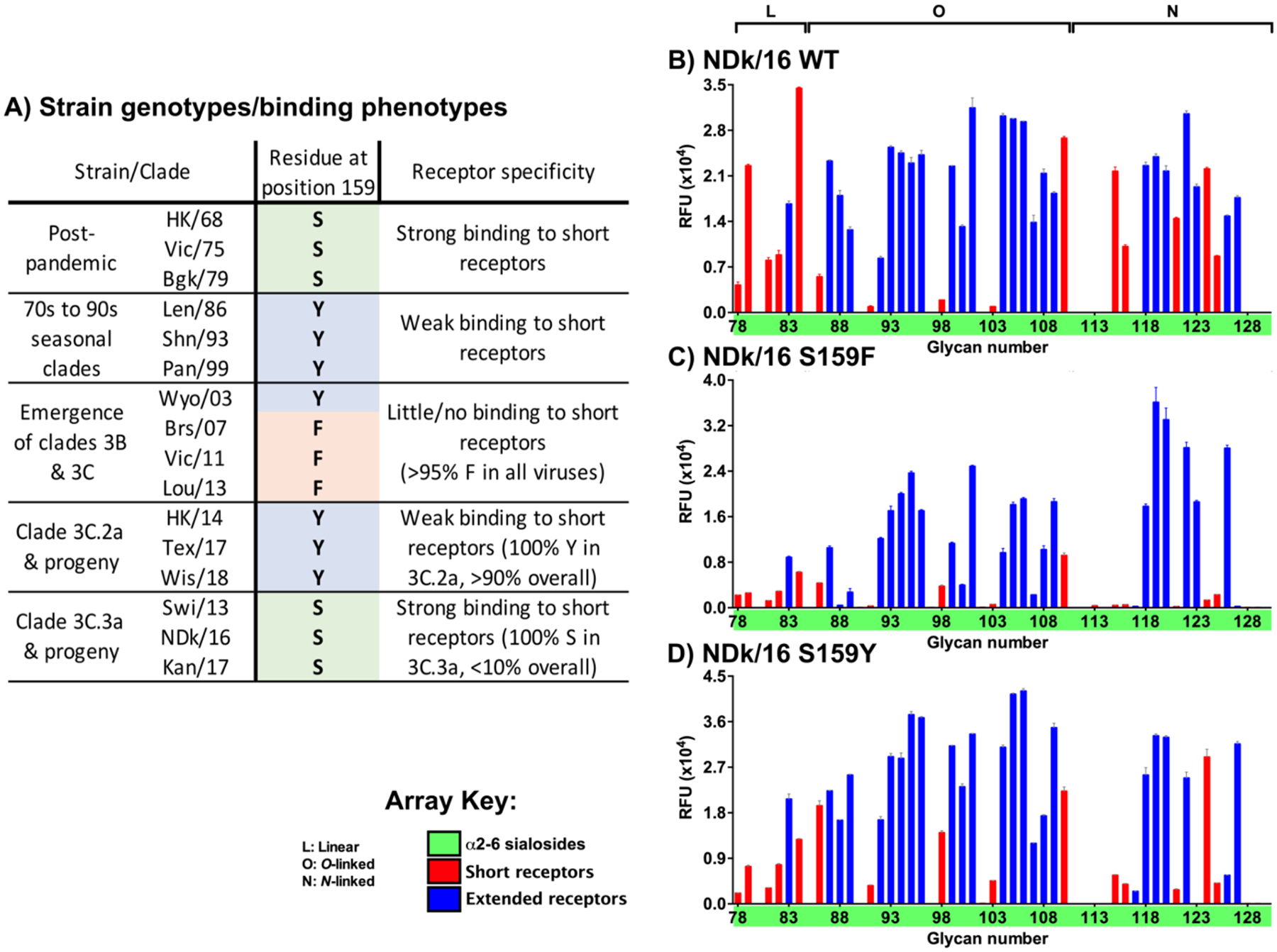
Correlation of length-dependent receptor binding and HA residue 159 (A) Summary graphic highlighting clades/strains utilized within this study, their receptor-binding specificity with regard to short vs extended glycans, and the identity of the amino acid at position 159 of HA. Interestingly, both within the clade 3C.2a/3C.3a split and historically, a bulky aryl-containing side chain (either tyrosine (Y) or phenylalanine (F) vs a small polar side chain (typically serine (S)) correlates strongly with length-selectivity. Panels (B – D) show engineering of a recombinant contemporary clade 3C.3a strain H3, A/North Dakota/26/2016, to alter its native receptor specificity. (B) WT NDk/16 shows equally strong binding to both long and short receptors, indeed, the most intense individual signal comes from receptor #84, the terminal trisaccharide fragment α2–6-sialyl-LacNAc (6SLN; see array V3 in Supplementary Data S1). Engineering of prior F159 (B) or contemporary (in clade 3C.2a) Y159 (C) either eliminates or strongly reduces binding to nearly all short receptors (see bars highlighted in red). Panels (B – D) depict data collected from Sialoside array versions, V3, V3, & V4, respectively; Receptor structures corresponding to glycan numbers for all array versions can be found in Supplementary Data S1. Full array plots for all HAs/variants shown are illustrated in Supplementary Data S2.

Utilizing HA amino-acid sequence alignments encompassing human H3N2 strains from emergence in 1968 through to some of the most recent strains available, including representatives from both clades 3C.**2a** & 3C.**3a** (see Supplementary Data S3), we sought to identify variant positions that might directly contribute to length-selective binding. Surprisingly, only one HA1 amino acid, position 159, stood out as a feature that correlated well with both the loss of to binding short receptors in post-pandemic strains, and reversion to binding short receptors in clade 3C.**3a**. Position 159 is present as a serine (S) in post-pandemic H3 HAs, evolving subsequently to a tyrosine (Y) or structurally similar phenylalanine (F) from the mid 1980s. It was retained as Y or F after that in the corresponding period where length-selective binding clearly arose (1990s), and suddenly reverting back in 2013 to a serine exclusively in clade 3C.**3a** viruses (Supplementary Data S3, Fig. 3A & S4B). As a direct test of the importance of residue 159 in restricting binding of short receptors, recombinant variants of the HA of a representative clade 3C.**3a** strain (A/North Dakota/26/2016) were converted from WT 159S to the major variants 159F and 159Y that are found in 3C.**2a** genotypes. Both the 159F and 159Y variants often resulted in strongly reduced binding to shorter sialoside receptors containing only one or two linear LacNAc repeats and a selective preference for elongated counterparts (compare binding to highlighted glycans in red bars, Fig. 3B – D). While the residue at position 159 is unlikely the sole determinant of receptor-binding length selectivity over the course of H3 evolution, the key role of a single amino acid as a meaningful contributor to receptor binding is consistent with known influenza biology, since the switch from avian- to human-type specificity in H1, H2 and H3 pandemic viruses has also been shown to be due to just one- or two-residue substitutions relative to their avian virus progenitors.^10,11,19,21^

### Contemporary H3N2 viruses exhibit significant expansion of receptor-binding residues

Although these data clearly highlight residue 159 as a possible key factor in length selectivity of human IAV H3 proteins, identification and confirmation of this position by the glycan microarray did appear somewhat surprising since it is clearly outside of the consensus RBS. To-date, key receptor-binding residues/contributors to binding specificity in nearly all influenza HAs have been located either within or in close structural proximity to the traditionally defined RBS, framed by the 130 and 220 loops and the 190-helix (see Fig. S2). Residue 159 is within the 150-loop, located next to and extending above the 190-helix that forms the upper surface of the RBS pocket. The 150-loop comprises part of antigenic site B (along with the 190-helix), one of the major antibody epitopes present on the upper surface of HA1 in the HA trimer.^28^ To determine if and how these distal residues can truly play a role in receptor binding and, in particular, to decipher any potential role in the evolution of length selectivity, we performed NMR analysis of glycan receptor binding in solution and obtained numerous HA-receptor crystal structure co-complexes across multiple H3 representatives from the past several decades.

**Figure 4.**
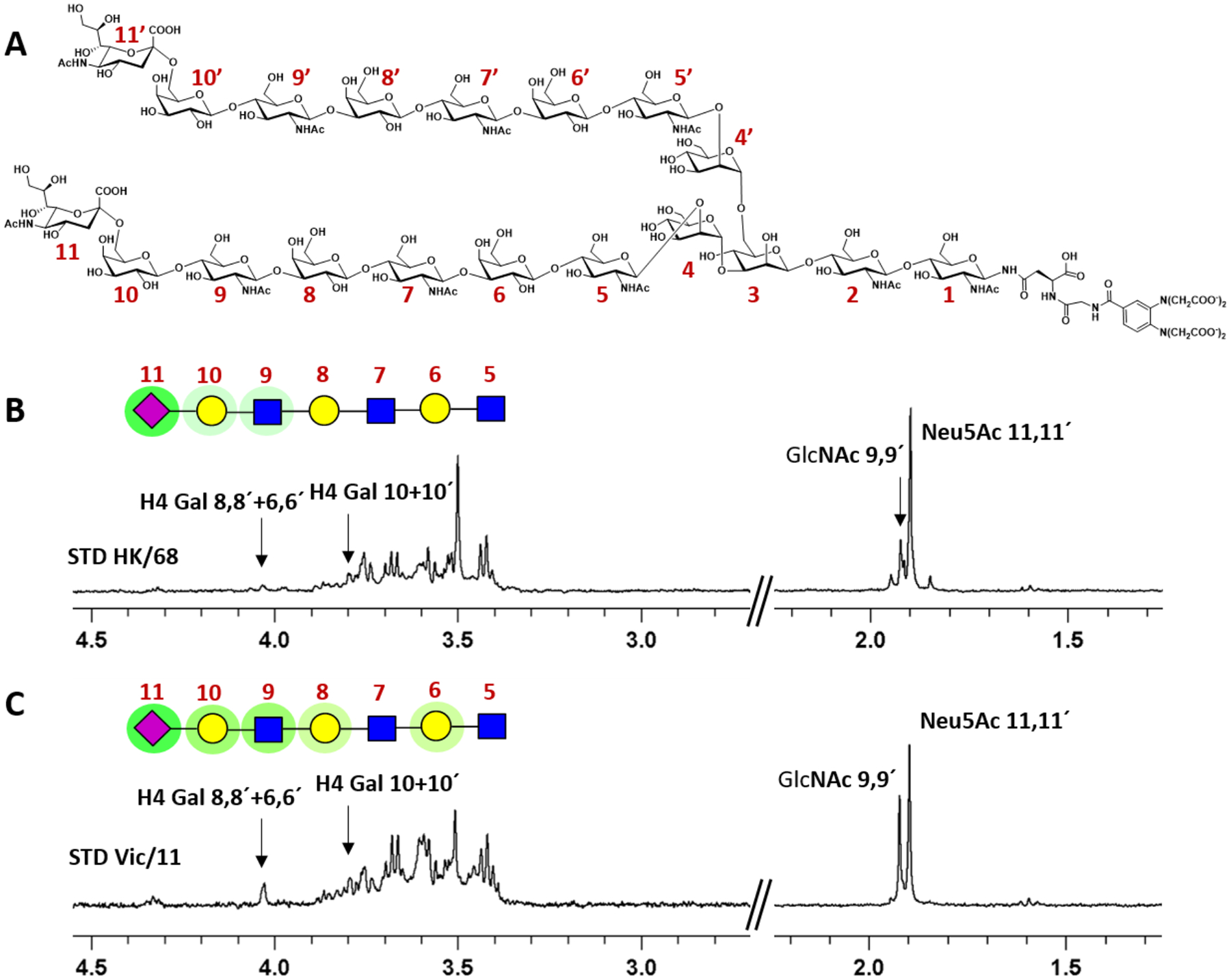
STD NMR analysis of early and late H3 receptor binding STD spectrum of a symmetrical, biantennary α2–6-sialylated N-glycan featuring three LacNAc repeats on each arm (6SLN_3_-N; panel A) interacting with either post-pandemic HK/68 recombinant H3 HA (B) or contemporary Vic/11 HA (C). Panel A shows the chemical structure of the synthesized glycan ligand, with individual sugar residues number from the reducing end through to Neu5Ac, duplicated residues on the 3’ or 6’ mannose branches are numbered X and X’, respectively. Panels (B & C) show a linear glycan cartoon of the terminal sugar moieties with interacting sugars highlighted in green. For HK/68 (B), binding is dominated by the strong and almost exclusive interactions of terminal Neu5Ac. For Vic/11 (C), binding interactions are clearly spread along the length of the receptor arm. A peak observed at approximately 3.5 ppm in both spectra (B & C) is residual solvent background from TRIS buffer and does not represent HA-glycan STDs.

STD-NMR (Saturation Transfer Difference Nuclear Magnetic Resonance) spectroscopy has become a powerful and extremely sensitive technique for analysis of small molecules, particularly glycans or small glycan fragments, interacting with (viral) receptor proteins in solution.^46,47^ Briefly, in STD experiments, magnetization is transferred from the protein to the glycan by intermolecular NOE (Nuclear Overhauser Effect). Saturation of specific resonances of the receptor is achieved by selective irradiation of a region of the spectrum (typically between 0 and −1 ppm) that contains shielded protein resonances but is free of glycan resonances; this spectrum is called “on resonance”. The saturation spreads rapidly from these resonances to the entire protein through intramolecular dipole-dipole interactions and transfers to any bound ligand. In a second experiment, an “off resonance” region free of any signals, is irradiated with the same pulse sequence to generate a reference spectrum. Finally, the on resonance spectrum is subtracted from the reference experiment. In this subtraction, we observe the signals of those protons that are directly involved in the interaction between protein and ligand, whereas signals of non-binding compounds are cancelled. The intensity of the STD effect is proportional to the proximity of the glycan protons to the protein. Here, we utilized the exceptional sensitivity of STD-NMR to probe binding of early-pandemic human H3 IAV HAs (minimal length selectivity for human-type receptors, see Figs. 1 & S1) compared with a representative contemporary H3 HA from clade 3C that exhibits among the strictest length-selective binding preferences we have observed to-date. Figure 4A shows the structure of a symmetric biantennary, α2–6-sialylated N-glycan with three LacNAc repeats beneath the terminal sialic acid on each arm (6SLN_3_-N). Panels B & C reveal STD-NMR analysis of this glycan binding to early (non-selective) and late (length-selective) recombinant H3 HA glycoproteins, with resonances corresponding to key protons in individual sugars highlighted by arrows. 6SLN_3_-N binding to HK/68 (A/Hong Kong/01/1968) H3 HA is clearly dominated by the major STD of the methyl group of Neu5Ac on the right of the spectrum (major peak at δ ~ 1.9 ppm), while the signal of the methyl groups of GlcNAcs 9,9’ appear with far lower relative intensity and the signals corresponding to H4 of internal Gal units (8,8’ & 6,6’) are barely detectable above the background noise. Contrastingly, binding of 6SLN_3_-N to Vic/11 H3 HA, while still strong for terminal Neu5Ac 11, reveals far stronger specific interactions with downstream sugars within the N-glycan branch, notably GlcNAc 9,9’ and internal Gal units 8,8’ & 6,6’. While the key proton resonances of the Gal 8,8’ and Gal 6,6’ overlap, recent STD NMR experiments using glycans with each Gal uniformily labeled with ^13^C_6_ show that Gal 6 interacts with the H3 HA of A//INFH-16-0019/2016, and that Y159 plays a substantial role in that interaction.^48^ Together, these data reveal that, even within a dynamic, solution-phase environment (as opposed to microarray analysis where candidate receptors are isolated onto a solid surface), contemporary human H3(N2) IAVs have evolved a far more complex pattern of substrate recognition/binding. Furthermore, the contribution of sugars distal to terminal sialic acid suggest elements located away from the classical RBS (such as residue 159) clearly contribute significant additional interactions along the length of a given glycan chain, favoring binding to extended sialoside receptors.

**Figure 5.**
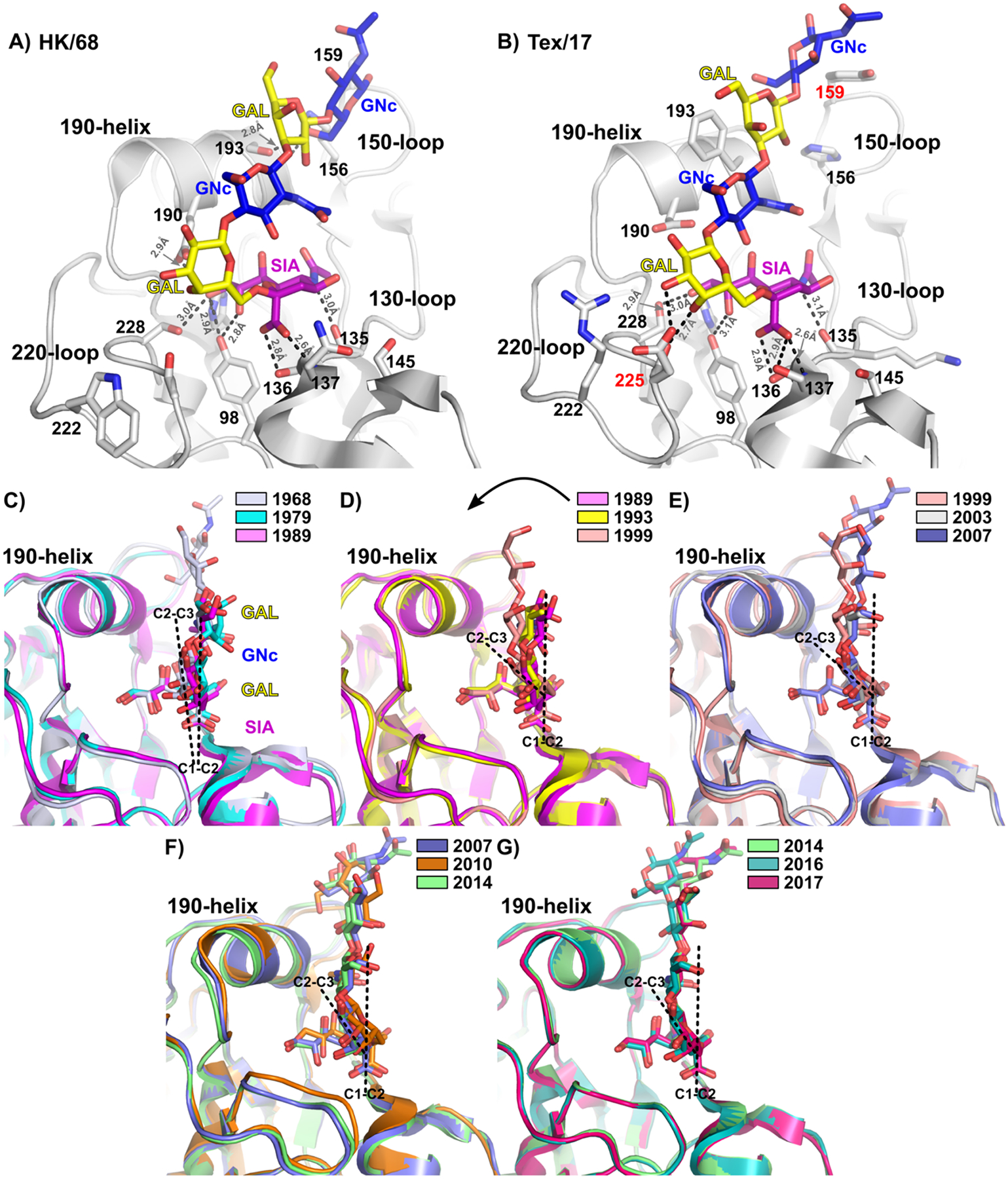
Crystal structure analysis of H3 receptor binding over time (A & B) Detailed structural views of α2–6-sialyl-(LacNAc)_2_ (6SLN_2_) bound to HK68 (early) and A/Texas/73/2017 (late; Tex/17) H3s HAs. Key secondary structural elements forming the RBS and amino acid side chains are labelled in black text (key evolved receptor-binding residues in panel (B) are highlighted in red), while receptor glycan moieties are labeled and highlighted according to CFG (Consortium for Functional Glycomics) color values. Panels (C – G) show triplet overlays of receptor-bound H3 HA structures progressing at intervals over the last five decades. Within each panel, the (invariant) approximately vertical angle of the C1-C2 bond of sialic acid is highlighted by a dashed black line, relative to the position of the sugar ring in the underlying galactose (highlighted by a second dashed line parallel to the C2-C3 bonds). As shown by an arrow in panel (D), between the late 1980s to late 1990s, the angle of the underlying sugar backbone undergoes substantial rearrangement to lie closer to the HA protein surface, highlighted by the C1-C2 vertical plane approximately bisecting the reducing end sugars in panel (C) compared to almost no receptor atoms lying on or over the same axis in panel (G).

To explore these interactions in more detail, we crystallized and solved the structures of seven H3 HA trimers, representing various time points across H3N2 evolution, in complex with either a terminal α2–6-sialylated LacNAc pentasaccharide (6SLN_2_, containing two LacNAc repeats) or trisaccharide (6SLN, containing a single LacNAc repeat). Together with four previously published structures, this panel represents a broad view of H3 receptor binding featuring a set of 11 receptor co-complexes at 5 – 10-yearly intervals beginning from 1968 through the following approximately 50 years of evolution (see Fig. S3 for a complete set of structures). Figures 5A & 5B (and Fig. S3A & S3K) show typical views of an extended 6SLN_2_ bound in the RBS of pandemic 1968 H3 HA (HK/68) and 2017 3C.**2a**-descended H3 HA (Tex/17), respectively. Interestingly, these static snapshots of receptor-binding across multiple decades in their crystallized form provide strong confirmatory evidence of data observed by solution-phase STD NMR experiments; i.e., that key H-bonding interactions in early post-pandemic H3 HAs are almost entirely focused on the terminal sialic acid moiety, whereas contemporary HAs appear to make additional interactions along the length of the receptor glycan chain, including evolved hydrophobic interactions (see below for details, Figs. 5 and S3). Crystal structures of HK/68, Bgk/79 (A/Bangkok/1/1979), and Bei/89 (A/Beijing/353/1989) HAs reveal a generally conserved pattern of either 7 or 8 H-bonds connecting N5 of SIA to the backbone O of residue 135, O1A of SIA to the Oγ of Ser136, O1B of SIA to the backbone N of residue 137, the side-chain OH of Tyr98 to O8 and/or O9 of SIA, and a combination of the side-chains of His183, Glu190, and Ser228 to O9 of SIA (Fig. 5). The structure of HK/68 HA in complex with 6SLN_2_ reveals just a single H-bonding interaction with a glycan residue other than SIA, suggesting a potential contact between the reducing-end GlcNAc (equivalent to GlcNAc 7/7’ in Fig. 4A) and Lys156 or Ser193. This feature does not appear to be well maintained since electron density for the final LacNAc repeat of 6SLN_2_ could not be observed in complex with Bgk/79 or Sh/93 (A/Shandong/9/1993) (Fig. S3B & D), or the terminal GlcNAc in complex with Mos/99 (A/Moscow/10/1999), while Bei/89 could only be solved in complex with the trisaccharide 6SLN (see Fig S3C; soaking with 6SLN_2_ resulted in unrecoverable crystal fracturing despite multiple attempts). Thus, while binding interactions along the length of glycan receptors potentially favoring selection of extended glycans do appear to occur in early H3 HAs, such interactions appear transient and poorly conserved. Co-complex structures of H3s from the 1990s onwards reveal an interesting pattern of redistribution of H-bonds around the terminal sialic acid moiety. While the overall number of direct contacts to SIA residues reduces only slightly, the focus of interactions appears to shift from the most buried portions of the sialic acid sugar within the RBS (i.e. hydroxyl groups at O8 & O9), where only contacts with Tyr98 and Ser228 are strongly maintained, to positions on the external face of the RBS more directly in contact with the NeuAcα2–6Gal linkage. Indeed, structures of Wyo/03 (A/Wyoming/3/2003), Bris/07 (A/Brisbane/10/2007), and Ecu/16 (A/Ecuador/1374/2016) HAs reveal interactions with O4 of SIA either directly, or through coordinated water molecules, and the evolution to Ser at position 137 from 1999 onwards (see alignment file in Supplementary Data S3) contributes an additional H-bond to O1B of SIA (Fig. 5B & S3E – K). However, from the late 1990s through early 2000s onwards, H3 HAs can clearly be seen to gain additional H-bonds, interacting more specifically with sugars beyond the terminal sialic acid, including the underlying galactose residue (Gal 10/10’ in Fig. 4; O3 & O4 interacting with Arg222 & Asn/Asp225, respectively), the central GlcNAc of 6SLN_2_ (GlcNAc 9/9’; N2 interacting with Asp190 via a conserved water observed in Bris/07, Mich/14 (A/Michigan/15/2014), and Ecu/16), and the reducing-end Gal (Gal 8/8’; making a direct H-bond via O2 to early H3s containing either Ser or Asn at position 193, or in contemporary examples stacking against the phenyl ring of the evolved Phe193 side chain, see Figures 5 and S3).

In addition to highlighting the expanding footprint and complexity of the H3 interaction with α2–6-sialoside receptors over the course of viral evolution, this collection of structures offers insights into the potential basis of length selectivity. Figures 5C – 5G present a series of triplet overlays of receptor-bound H3 structures (viewed side-on) focusing on the bound receptor position, relative to HA over time. Here, structural alignments were conducted using only the head domain of respective HA1 subunits, including the coordinates of the bound sialic acid moiety as a fixed reference point. In all cases [with the possible exception of Minn/10 (A/Minnesota/11/2010) where both SIA and the 220-loop appear slightly displaced from all other structures, see Fig. 5F], alignment of RBS elements in structures closely related in time/evolution produces close structural matches with bound sialic acids and key secondary structure elements occupying very close or essentially invariant positions (Fig. 5C – G). In each panel, linear planes, represented by broken black lines, highlight the relative positions of the axial C1-C2 bond in the SIA moiety of the latest depicted structure (except for 5C where 1968 is highlighted), relative to the plane of the underlying GAL sugar ring (depicted by a line parallel to C2-C3 bonds). Throughout, positioning of the C1-C2 axis of SIA is largely fixed and appears close to vertical. However, the plane corresponding to atoms within the Gal sugar ring undergoes a substantial rearrangement throughout H3 evolution. Initially, in Fig. 5C, atoms within the GAL sugar ring appear to lie within 10° of the vertical (illustrated by C2-C3 bond) created by the C1-C2 SIA plane, and thus the reducing-end sugars in the respective chains make minimal interactions with HA and are positioned facing out of the RBS toward solvent space (note particularly the large portion of ligand atoms in Fig. 5C that cross (to the right of) the vertical SIA C1-C2 line toward the right side of the panel). Figure 5D shows that between 1989 and 1999, approximately in-line with rearrangements in H-bonding observed around the terminal SIA residue, a major shift occurs in the relative position of underlying sugar residues. The two LacNAc repeats observable in 6SLN_2_ complexes shift to a position in considerably closer proximity to HA side chains within the 190-helix and 220-loop secondary structural elements. Importantly, this alteration in the positioning of LacNAc sugar residues (a change equivalent to approximately a 30 – 40° shift the positioning of the GAL sugar ring relative to SIA, see C2-C3 bond) appears to have become permanent, with bound ligand conformations observable in essentially matched positions through the following approximately 20 years. The crystal structures are consistent with solution-phase STD NMR binding analysis (Fig. 4), which revealed a substantial buildup of interactions between HA and the receptor beyond the terminal sialic acid, and an apparent trend towards more complex binding from 1968 to 2011 H3 HAs.

### The structural basis of length selectivity and the role of residue 159

While our observations surrounding the amino acid at residue 159 are consistent and predictive of H3 receptor binding preferences by glycan length, it is clear from structural data that evolution through the late 1980s to 1990s is complex and encompasses residues within the canonical secondary structure features that make up the RBS (see Figs. S2 & S3). The alteration in receptor binding conformation shown in Figures 5C – G is principally contributed by HA variation in the 130- and 220-loops making new H-bonds with galactose and sialic acid. Residues 190 and 193 no longer make H-bonds around the O9 of sialic acid and the second GlcNAc, adapting instead to make new interactions along the sugar chain and giving rise to a slight structural shift in the overall positioning of the 190-helix.^49^ Taken together, these observations suggest a dual mechanism of adaptation or control over selection for extended glycans. Firstly, between 1968 and 2000, enhanced binding to longer receptors evolved by spreading binding interactions along the length of the receptor sugar chain, rather than concentrating mainly on the terminal sialic acid moiety or terminal sugar linkage. Subsequently, the evolved binding mode of receptor glycans appears to have become fixed, even in the face of the evolution of clade 3C.**2a**- and 3C.**3a**-descended viruses which, as noted above, respectively conserve and reverse their exclusive selection for extended receptors.

Within these contemporary strains, receptor length selectivity appears to be strongly influenced by single variants at position 159. Comparison of complex structures between Ecu/16 and Tex/17, respective representatives of clades 3C.**3a** and 3C.**2a**, reveals a potential role for residue 159 occupying a space in close proximity to the reducing end of the 6SLN_2_ receptor fragment above the RBS (Fig. S5). As described, when present as S159, H3 viruses bind similarly to both long and short receptors; however, when present as the alternate commonly observed variants Y159 or F159, the otherwise closely similar HAs bind preferentially to elongated glycans (Fig. 3). Analysis of the Ecu/16 (S159) structure reveals that the smaller serine side chain creates substantial additional space at the top of the HA head domain, allowing free rotation of the reducing-end GlcNAc (compare the turquoise receptor in Fig. 5G and S5). Alternatively, Mich/14 and Tex/17 structures (both Y159) appear comparatively rigid, with the terminal GlcNAc occupying a similar position to receptor complexes from 2007 & 2010 featuring F159 (Fig. S5G & H). In these cases, the presence of the larger phenylalanine side chain not only adds additional steric bulk at the top of the HA head, but further forms a conserved stacking interaction with the sugar ring of GlcNAc 7 (Fig. 4A numbering). Such an interaction appears to favor binding of longer receptor species. While a structural explanation for this phenotype is not immediately obvious, it seems possible that the additional space created by S159 promotes binding to short receptors since sugar residues underlying the equivalent of GlcNAc 7 consist of bulky branchpoint trisaccharides (in N-glycan and certain O-glycan cores) or GalNAc attached directly to an amino acid (majority of linear O-glycan cores). Consistently, F/Y159-containing species favor binding to longer receptors since additional linear sugars in the receptor chain are potentially capable of extending beyond the 150-loop to avoid steric hindrance.

### Evolution of the length selectivity trait from 1968 pandemic strains represents an epistatic adaptation to human hosts

One of the hallmarks of evolution/adaptation of an organism to a new environment is the fixing of newly acquired phenotypic features within subsequent generations, where key novel traits become irreversible even within the context of subsequent downstream evolution. To test whether the alterations in receptor binding interactions and conformation observed between the late 1980s and early 2000s H3 HAs represents a true evolutionary shift, we performed a large-scale mutational analysis to determine how easily reversible this phenotypic shift might be. Since co-complex crystallization and direct structural observation of potentially tens of H3 variants would likely prove both extremely costly and time consuming, we instead chose to screen for reversal of the length-selective binding phenotype via glycan microarray analysis, which is a technique far more compatible with high-throughput sample generation and analysis. To avoid confounding results from strains where length-selectivity appears switchable based on addition or removal of bulky side chains at position 159, as documented for clades 3C.**2a** & 3C.**3a**, we selected an earlier clade 3C strain, Vic/11, as a study model. Here, our strategy was to create large numbers of structure-informed point mutants, reversing evolved receptor-interacting side chains back to their prior identities within the 1968 pandemic strain HK/68. The HK/68 and Vic/11 ectodomains comprise a total of 71 variant positions between them (Supplementary Data S3). We created either single point mutants or combinations of variants from 19 key glycan-interacting (or closely proximal) H3 amino acid positions, including 142, 145, 156, 157, 158, 159, 160, 163 186, 188, 189, 190, 192, 193, 196, 222, 225, 226, & 227, totaling over 140 individual expressed protein constructs. Many single-variants and a minority of double- or triple-mutant combinations retained receptor-binding function, all with little or no impact on length specificity. However, larger variant combinations, including a 13-mutant construct (Vic/11-R142G-N145S-H156K-L157S-N158G-F159S-K160T-A163V-F193S-A196V-R222W-N225G-P227S) all failed to reconstitute comparable binding to the WT HK/68 construct (see Fig. S6 for a summary of positive binders and Supplementary Data S2 for full glycan array results). These results document an exceptionally high genetic barrier to reversion of receptor-binding specificity and conformation, likely requiring near wholesale reversal of most variant positions including potentially some at positions quite distal to the RBS. These findings explain the changes in receptor binding that become fixed during evolution and are consistent with the observed long-term adaptation of H3 viruses to human hosts.

### Receptor binding in contemporary H3N2 viruses is linked to antigenicity

**Figure 6.**
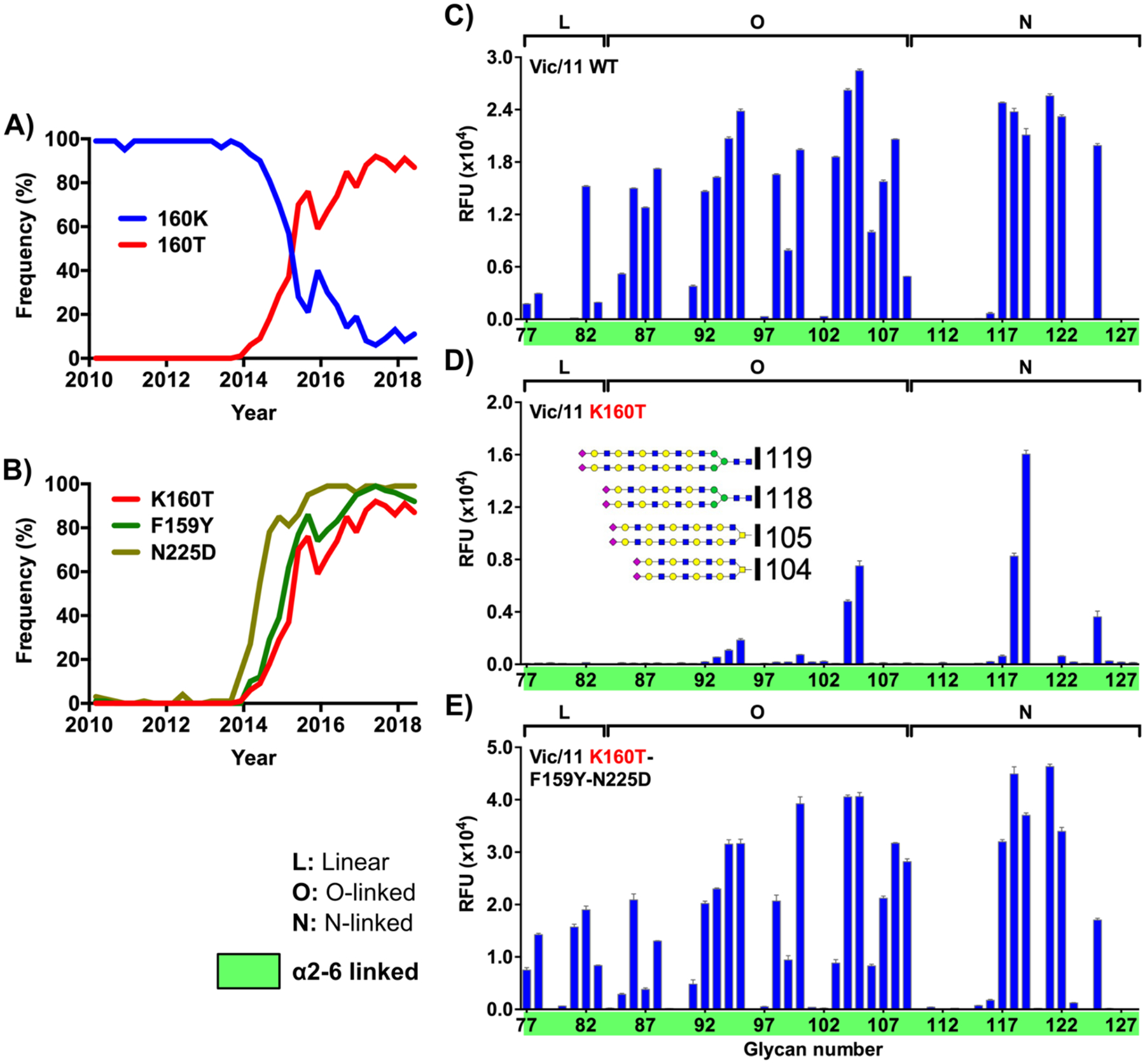
Receptor binding variants allow evolution of a novel glycan via K160T Selection of a novel K160T variant in clade 3C.2a H3N2 viruses from 2014 onwards led to formation of a new glycan (at N158) within the head of H3 HAs (A). Engineering of K160T within strains prior to the emergence of clade 3C.2a shows the novel glycan to be deleterious to receptor binding (C & D), with novel variants only able to bind the most extended receptors featuring 4 or 5 LacNAc repeats (D, shown inset). Sequence analyses reveal two functionally linked receptor-binding variants emerging immediately prior to K160T (B) restore native receptor-binding function when combined with the this new variant (E). Panels (C – E) depict data collected from Sialoside array versions, V1, V3, & V5, respectively; Receptor structures corresponding to glycan numbers for all array versions can be found in Supplementary Data S1. Full array plots for all HAs/variants shown are illustrated in Supplementary Data S2.

Given the demonstrated role in receptor binding and recognition of residues in the 150-loop of H3, particularly within the contemporary 3C.**2a** & 3C.**3a** H3 clades, we were interested to investigate any potential effects on antigenicity. The 150-loop forms part of antigenic site B and variants emerging within this region are most commonly associated with antigenic drift in response to immune pressure.^50^ Exchange of small polar side chains for large aromatic side chains, such as S159 to F/Y159, not only influence receptor binding as demonstrated here, but potentially also affect antibody responses. Thus, we sought to investigate the effect of recent antigenic changes within this region. Beginning in mid-2014, a majority of viruses in clade 3C.**2a** began to emerge with a K160T variant (Fig. 6A), creating a novel N-glycosylation sequon (N-X-S/T) by virtue of a conserved upstream asparagine at position N158.^37,50^ Emergence and gradual dominant spread of this novel glycosylation site led to a dramatic change in receptor binding, as evidenced by poor hemagglutination of erythrocytes^50^ and poor growth in eggs,^37,51^ presumably due to lack of extended glycans on erythrocytes and the chorioallantoic membrane. Poor growth in eggs thus subsequently led to a vaccine mismatch due to strong selective pressure on variants without a glycan at N158, that likely become positively selected for owing to permissive binding to short glycan receptors in this host. Lack of a glycan in strains administered as vaccines generated additional, and often immunodominant epitopes, inducing host antibodies that did not bind to the circulating seasonal viruses in the following influenza season.^37^

While a common antigenic adaptation in avian HAs, particularly H5s,^52^ where the α2–3 sialosides are extended and approach more laterally to the RBS, the emergence of such a glycan variant was perhaps unexpected in human viruses. The added glycan, while useful in successfully masking antibody epitopes, creates additional steric bulk at the top of the head domain that could potentially occlude incoming receptor glycans/glycoproteins. Indeed, when we introduced K160T into Vic/11 HA, we observed substantial inhibition of receptor binding, with only the very longest receptors (containing 4 or 5 LacNAc repeats) present on the glycan microarray possessing sufficient size and/or flexibility to overcome this novel glycan addition (Fig. 6C & D). Despite this finding, array data for post-2014 HAs and whole viruses studied here (see Fig. 1 & Supplementary Data S2) reveal no such apparent disruption, with all samples analyzed maintaining comparable specificity patterns to both prior and contemporary strains without the N158 glycan.

Analysis of sequence alignments, similar to that performed above to identify the role of residue 159 in length selectivity of contemporary viruses, revealed that two major (and subsequently dominant) RBS-proximal variants, N225D and F159Y, arose immediately prior to, or concomitant with, the evolution of K160T in clade 3C.**2a** (Fig. 6B). Reconstitution of these two closely occurring mutations within the Vic/11-K160T background restored the robust binding phenotype of native K160T-carrying H3 HAs (Fig. 6E). Since alternative variants at residue 159 have already been shown to play a deterministic role in length selectivity between H3s in clades 3C.**2a** and 3C.**3a**, we sought to dissect the individual contributions to novel glycan evolution of both the 159 and 225 species (Fig. S7). While the Vic/11-K160T-F159Y double variant does somewhat restore binding functionality, glycan microarray analysis shows an atypical receptor specificity pattern with weak binding to short α2–6-sialoside receptors and some binding to α2–3-linked receptors, which are highly uncharacteristic of either the parent Vic/11 or later clade 3C.**2a** strains (Fig. S7A). Alternatively, pairing of Vic/11-K160T with N225D reveals an almost entirely native-like binding phenotype, which was similar to both native H3 viruses and a Vic/11 triple mutant (compare Fig. S7B & 6E), indicating that this variant is most likely the dominant driver permitting evolution of the K160T variant and novel glycan acquisition. Interestingly, however, it should be noted that while N225D undoubtedly appears to be the major contributor, this variant emerged and became dominant across both clades 3C.**2a** and 3C.**3a**, while both K160T and F159Y are exclusively found together and only within clade 3C.**2a** viruses, suggesting a potentially more subtle yet still-unresolved link between the two. For example, since Y159 can interact directly with Gal 6,6’ units within extended glycans,^48^ it may serve to help ‘guide’ the extended chain past the glycan at N158 below the bulk of the branched glycan unit.

That N225D appears a major driver of a recent substantial antigenic shift in H3N2 viruses is both surprising and interesting, since it more strongly links properties of receptor-binding and antigenicity than initially expected. Indeed, residues at position 225 are physically distant from antigenic site B and almost solely associated with receptor binding (variants at positions 225 and 190 underly the specificity switch contributing to emergence of H1N1 viruses in humans^18^). Furthermore, the structural effects of N225D are not obvious since both side chains are isostructural and, as noted above, both N225 & D225 variants appear to make identical interactions with the penultimate galactose underlying sialic acid (Fig. S3G & I – K). However, it remains possible that a potential full negative charge present on the basic aspartate (D) might well contribute stronger electrostatic interactions than the polar asparagine (N). Analogously, when inserted into Vic/11 150-loop variant backgrounds that initially appeared to abolish specific native binding on glycan microarrays, N225D successfully restored at least some, up to most, functional receptor engagement for a number of mutants (Fig. S7C). Together, these data represent a paradigm in modern H3 HAs where certain receptor-specific variants that appear to enhance binding, particularly at position 225, can directly promote or repress selectable variants within more distal and antigenically relevant sites.

### Contributions of the NA to evolution of receptor specificity.

**Figure 7.**
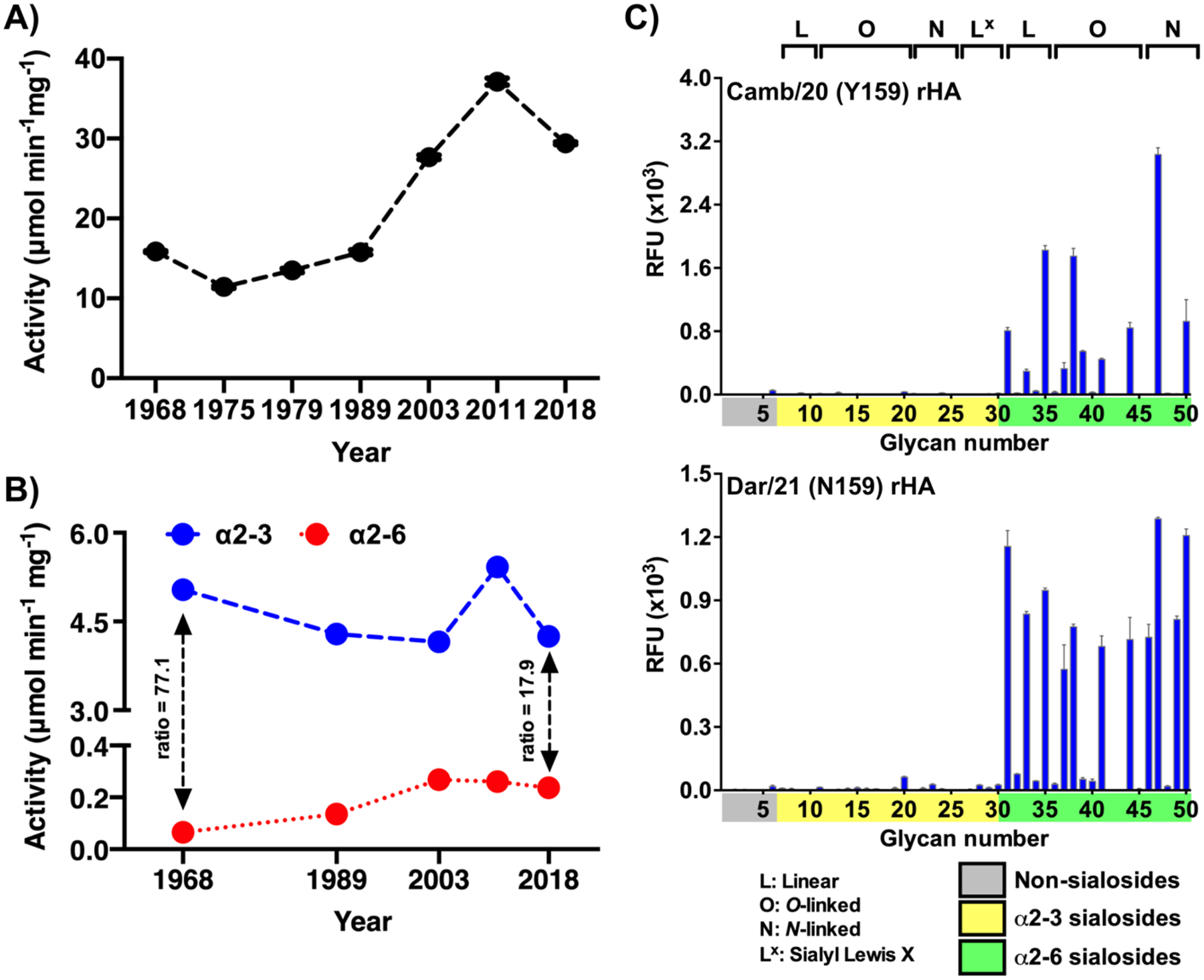
Evolution of NA activity and a novel 159N HA variant (A) Absolute N2 activity, as measured against the optimized fluorescent substrate, MUNANA, steadily increases over time in line with evolution in H3 receptor binding. (B) Concurrently, within a subset of the same panel of N2 representatives, specific activity measured against 2–3- or 2–6- linked NeuAcα-Gal-pNP substrates reveals a long-term narrowing trend in substrate preference between avian- and human-type receptors, suggesting slow evolution away from the avian origin of these enzymes. (C & D) Glycan microarrays showing the effects on receptor specificity of a recent Y159N variant emerging in clade 3C.**2a** viruses during 2021. A prior strain, Camb/20 (A/Cambodia/e0826360/2020) maintaining the canonical Y159 shows selectivity for extended α2–6 receptors with at least 3 LacNAc repeats, while the newly evolved Dar/21 (A/Darwin/6/2021) possessing N159 binds species containing 2 or more LacNAc repeats.

Finally, while HA remains a major determinant of host adaptation, it is well established that properties of specificity and binding avidity for glycan receptors are closely linked with the corresponding activity of NA. IAV NAs function in concert with HA to release newly formed virus particles from the same receptors on the surface of host cells, as well as avoiding unfavorable interactions with extracellular mucins or even other virus particles, through a concept known as HA-NA balance.^53,54^ Thus, as H3 HAs evolve under immune selective pressure, while maintaining fitness to bind host receptors on human cells, corresponding changes in NA activity are required to maintain that balance. We described above that human H3 HAs have steadily evolved to selectively recognize extended glycans and to bind to them with higher avidity.^26^ To determine if N2 activity follows a similar trend, we initially profiled absolute (i.e. linkage-independent) activity against a panel of recombinant NAs from 1968 through to contemporary viral strains against the widely-used fluorescent reporter MUNANA (4-Methylumbelliferyl-N-acetyl-α-D-Neuraminic Acid). As shown in Figure 7A, as overall adaptation to human receptors promoted increasingly strong and complex HA receptor binding, total NA activity also steadily increased, presumably to facilitate release of more tightly bound progeny virions and maintain overall HA-NA activity balance.^53,54^ However, while MUNANA activity yields a convenient and simple overview of NA activity, deployment of an artificial leaving group lacking the authentic linkage chemistry of a native saccharide moiety overlooks key information regarding specific adaptation to α2–6-linked sialosides that form the major receptor structures on the human airway. To address this issue, we utilized a linked enzyme activity assay featuring recombinant N2 NAs combined with a bacterial galactosidase, to assess activity against native α2–6- or α2–3-linked NeuAcα-Gal disaccharide substrates with a chromogenic leaving group, para-nitrophenol (pNP). Here, activity of galactosidase (present in large excess) is blocked until the non-reducing end sialic acid sugar is removed by NA, giving an indirect but linkage-specific readout of NA activity since the viral enzyme represents the rate-limiting step. Analysis of a subset of our recombinant N2 panel reveals that, over decades of evolution, specific activity against human α2–6-sialoside receptors has also been steadily increasing. Long-term α2–3-specific activity inherited from the avian origin of these genes has gradually decreased, with the ratio of α2–3:α2–6 activity (measured in μmol min^−1^ mg^−1^) decreasing from 77.1 ± 0.01 in 1968 to 17.9 ± 0.06 in 2018, an effective 4-fold adaptive alteration (see Fig. 7B). While H3N2 IAVs are undoubtedly strongly established within the human population, together, these data characterizing the two major viral-surface glycoproteins paint a picture of a population still evolving within its host environment, albeit over a comparatively lengthy time scale, and still successfully selecting for variants with enhanced fitness and capacity to infect humans and cause disease.

### Implications of evolved receptor specificity for IAV vaccines.

Most contemporary H3N2 IAV strains from the last two decades exhibit exclusive binding to extended α2–6-linked sialosides yet retain their ability to circulate within the human population, and even dominate over clades and strains with more broad specificities. A similar preference of H1N1 viruses for extended glycans has also been noted, but appears less extreme as for H3N2 viruses, where binding has been reported to α2–6 sialylated glycans with two LacNAc repeats.^55,56^ These observations suggest that extended glycan receptors are present on cells within the human airway. In this regard, extended N-linked glycans have been documented on a human epithelial cell line,^56^ in lung tissues,^22^ in the glycome of whole lung,^23^ and within ferret respiratory tissues^57^.

We view the origin of this restricted specificity for extended glycans to be a consequence of immune selection to evade existing human immunity while simultaneously maintaining fitness for airway receptors. A practical consequence of the restricted specificity for extended glycan receptors is that the virus has had a diminished ability to grow in embryonated hen eggs used for the production of vaccines.^24,58,59^ Glycome analysis of egg membranes has revealed that N-linked glycans are partially capped by α2–6 sialic acids, but have only one LacNAc repeat.^60,61^ This accounts for the very poor growth of recent H3N2 viruses in eggs. As a result, during virus production in eggs, receptor variants that bind better to egg membrane receptors for better growth can also have altered antigenic properties that can impact vaccine efficacy.^62,63^ A real-world example of this can be noted in seasonal H3N2 influenza strains emerging from 2014 onwards. During this period, emergence of the N158 glycan (Fig. 6) led to substantial vaccine mismatching since strains without this novel feature were initially selected for vaccine production.^37,50^ Since then, data have shown that, even when correctly selected, N158-glycosylated strains grow poorly in eggs leading to rapid glycan loss at this position^51^. The US CDC data have revealed enhanced disease burden and poor vaccine effectiveness in all seasons where H3N2 strains have dominated since this time^59,64^. Recent results suggest that MDCK cells produce extended glycan receptors^60,65^ and, when enhanced with the sialyltransferase ST6Gal1 that produces ‘human-type’ receptors, there is less selection of receptor variants.^40,66^

For H3N2, since 2020/21, new strains with a previously unknown N159 variant have begun to emerge and become dominant. Interestingly, N159-containing HAs show an intermediate length-selective phenotype, binding specifically to receptors containing two or more LacNAc repeats, similar to H1N1 viruses. However, given the reduced levels of influenza circulation in humans over recent seasons, it remains to be seen if this variant will continue to be selected in the long-term or what effects it may have on vaccine effectiveness. Current advances in vaccine technology, including examples of both nucleic acid and protein subunit designs against SARS-CoV-2, where the sequence of the underlying immunogen is no longer dependent on replication in unnatural cells/cell line hosts, may well prove key to overcoming vaccine mismatch with seasonal H3N2 immunization. Ultimately, what is important to the influenza virus is to maintain the ability to bind host cell glycans as receptors and for transmission in the human population, a property that H3N2 continues to demonstrate strong capacity for. Constant surveillance of the evolution of receptor specificity of influenza virus will continue to have relevance to the propagation of viruses in eggs and mammalian cells to prevent selection of antigenic variants that reduce vaccine efficacy.^62,63^

## STAR METHODS:

### RESOURCE AVAILABILITY

#### Lead Contact And Materials Availability:

Further information and requests for resources and reagents should be directed to and will be fulfilled by the Lead Contact, James C. Paulson (jpaulson@scripps.edu).

Any unique reagents generated in this study are available from the Lead Contact with a completed Materials Transfer Agreement.

### EXPERIMENTAL MODEL AND STUDY PARTICIPANT DETAILS

#### Cell Cultures:

HEK 293T cells (human embryonic kidney cells, female) were maintained in DMEM medium supplemented with 10% fetal bovine serum (FBS), and 100 U mL^−1^ of Penicillin-Streptomycin. MDCK cells (Madin-Darby canine kidney cells, female) were maintained in MEM medium supplemented with 10% FBS, 2 mM L-Glutamine, and 100 U mL^−1^ of Penicillin-Streptomycin. MDCK-SIAT1 cells (Madin-Darby canine kidney cells with stable expression of human 2,6-sialyltransferase, female) were maintained in MEM medium supplemented with 10% FBS, 2 mM L-Glutamine, 100 U mL^−1^ of Penicillin-Streptomycin, and 1 mg mL^−1^ G418 sulfate.

#### Influenza virus:

Live influenza virus seed stocks were obtained from the International Reagent Resource (IRR; www.internationalreagentresource.org) and grown in MDCK or MDCK-SIAT1 cultures (approximately 40 million cells per virus) in MEM medium supplemented with, 2 mM L-Glutamine and 100 U mL^−1^ of Penicillin-Streptomycin. Cell cultures were washed twice in warm PBS prior to the addition of virus, typically diluted 1:1000 in growth medium. Diluted virus was incubated with cell cultures for 1 hour before being removed and replaced with growth medium supplemented with 2 μg mL^−1^ tosyl phenylalanyl chloromethyl ketone (TPCK)-trypsin and further incubated for 72 hours. At day 3, culture supernatant was recovered and centrifuged at 1000x g to remove cell debris before final centrifugation at 65,000x g for 2 hours at 4°C to isolate viral pellets. Viral pellets were resuspended in 1 mL PBS supplemented with 5% (w/v) sterile glycerol, aliquoted, and stored at −80°C. For measuring virus titer by the TCID_50_ (median tissue culture infectious dose) assay, MDCK or MDCK-SIAT1 cells were washed twice with PBS prior to the addition of virus, and viral growth medium supplemented with 2 μg mL^−1^ TPCK-trypsin. For measuring viral HA titers (median hemagglutination), virus stocks were diluted in PBS and an equal volume of 0.5% turkey red blood cells added.

### METHOD DETAILS

#### HA expression and purification for glycan microarray and NMR analysis:

Genes encoding HA ectodomains (residues 11–521 (see Supplementary Data S7); H3 numbering) were cloned into a customized DNA vector for expression in mammalian tissue culture featuring an N-terminal CD5 signal peptide, a C-terminal leucine zipper (GCN4) motif, and His8-tag using the NEBuilder HiFi DNA Assembly Master Mix. Final expression constructs were transfected into HEK293T cells using linear PEI (polyethylimine) at 5:1 w/w. After 12 hours, transfected cells were exchanged into serum-free media and incubated for a further 48 hours at 37°C, 5% CO_2_. Recombinant HA trimers were purified directly from condition media by IMAC using a 1 ml HisTrap FF crude column (GE). HAs were eluted in a gradient of PBS containing 0.5 M (final) imidazole, washed, and concentrated to 0.5 – 1.0 mg ml^−1^ final stock.

#### HA expression and purification for crystallization, crystal screening, & ligand soaking:

HAs were expressed and purified as described for Vic11 HA^69^ and were concentrated to 10 mg mL^−1^. Initial crystal screening was carried out using our high-throughput, robotic CrystalMation system (Rigaku, Carlsbad, CA) at Scripps Research. Initial crystal screening was based on the sitting drop vapor diffusion method at 4°C and 20°C with 35 μL reservoir solution and each drop consisting of 0.1 μL protein + 0.1 μL precipitant. Further refinement of the conditions obtained from the initial hits was performed manually by the sitting drop vapor diffusion method with 500 μL reservoir solution and each drop consisting of 0.8 μL protein + 0.8 μL precipitant. The crystallization conditions were as follows: Bgk/79 (A/Bangkok/1/1979) H3 HA: 40% PEG-600 and 0.1 M CHES pH 9.5 at 20°C; Bei/89 (A/Beijing/353/89) H3 HA: 1.0 M Li chloride and 10% PEG-6000 at 20°C; Sh/93 (A/Shandong/9/1993) H3 HA: 40% PEG-400 and 0.1 M imidazole pH 8.0 at 20°C; Mos/99 (A/Moscow/10/1999) H3 HA: 40% PEG-400 and 0.2 M Ca acetate, 0.1M HEPES pH 7.5 at 20°C; Mich/14 (A/Michigan/15/2014) H3 HA: 40% PEG-400, 0.2 M Li sulfate, and 0.1M Tris pH 8.5 at 4 °C; Ecu/16 (A/Ecuador/1374/2016) H3 HA: 50% PEG-200, 0.05 M Li sulfate, and 0.1M Tris pH 7.0 at 20 °C; Tex/17 (A/Texas/73/2017) H3 HA: 30% 1,2-propanediol, 20% PEG-400, and 0.1M HEPES pH 7.5 at 20°C.

For Bei/89 HA, crystals were soaked in reservoir solution supplemented with 15% ethylene glycol (cryoprotectant) and 6SLN for 2 hours. For other HAs, crystals were soaked in reservoir solution supplemented with 20 mM of 6SLN_2_ for 2 hours. The resulting crystals were flash cooled and stored in liquid nitrogen until data collection.

#### HA structure determination with bound ligands:

Diffraction data on HA-ligand complexes were collected at the APS GM/CA-CAT 23ID-B, 23ID-D, and at the Stanford Synchrotron Radiation Lightsource beamline 12-2. The data were indexed, and integrated and scaled using HKL2000^70^ (HKL Research). The structure was solved by molecular replacement using Phaser^71^ with PDB: 4FNK^72^ as the molecular replacement model, the structure was rebuilt using Coot,^73^ and refined using Refmac5^74^ and PHENIX.^75^ Ramachandran statistics were calculated using MolProbity^76^ (see Supplementary Data Table S4).

#### NA expression for enzyme kinetics:

Genes encoding NA ectodomains (residues 37–469; N2 numbering) were cloned into a customized DNA vector for expression in mammalian tissue culture featuring an N-terminal CD5 signal peptide, His8-tag, and a tetramerization domain from human vasodilator-stimulated phosphoprotein (VASP)^77^ using the NEBuilder HiFi DNA Assembly Master Mix. Final expression constructs were transfected into HEK293F cells using linear PEI (polyethylimine) at 5:1 w/w. Transfected cells in suspension were incubated for 6 days at 37°C, 80% humidity, 8% CO_2_. Recombinant NA tetramers were purified directly from media by IMAC using a 1 ml HisTrap FF crude column (GE). NAs were eluted in a gradient of 20 mM Tris-HCl (pH 7.0), 200 mM NaCl buffer containing 0.5 M (final) imidazole, washed, and concentrated to 0.2 – 0.5 mg ml^−1^ final stock.

#### Glycan arrays:

Recombinant HA trimers (50 μg ml^−1^ final) were pre-complexed with the anti-His mouse antibody (Thermo Fisher Scientific) and the Alexa488-linked anti-mouse IgG (Thermo Fisher Scientific) at 4:2:1 molar ratio for 15 min on ice in 50 μl PBS-T. This complex was incubated on the array surface in a humidified chamber for 60 min before washing and analysis. For GNL detection of whole viruses, virus stocks diluted to 256 HAU (final) in PBS, 3% BSA were incubated on the array surface in a humidified chamber for 60 min, followed by washing and incubation with either GNL:Streptavidin-Alexa488 for a final 60 min. Following final washing, all arrays were scanned using an Innoscan 1100AL microarray scanner (Innopsys). A complete list of the glycans on the array is presented in Supplementary Data S1, glycan schematics/cartoons shown are according to the Symbol Nomenclature for Glycans recommended by the NLM.^67,68^ Fully processed glycan array plots are presented in Supplementary Data S2, while full descriptions of the microarray experiment and datasets are presented in Supplementary Data S5 and S6 according to the MIRAGE consortium format.^78,79^

#### STD-NMR analysis:

NMR samples were prepared in deuterated TRIS buffer (D_2_O, hexadeuterated TRIS 50 mM, 50 mM NaCl, pD 7.8) to a final protein:glycan ratio of 1:61 (4.9 μM: 300 μM). STD experiments were acquired at 293 K with an on-resonance frequency of −0.3 ppm and an off-resonance frequency of 100 ppm on a Bruker AVANCE 600 MHz spectrometer equipped with a cryoprobe. The saturation time was 2 s. The triLacNAc biantennary glycan, used in STD experiments, was synthesized with a lanthanide binding tag (to test paramagnetic experiments). However, the STD spectra have been acquired in the absence of metal. In a parallel work, NMR experiments were acquired in the presence of a paramagnetic ion (Dy^3+^)^80^; however, the flexibility of the peptide linker between the chelating unit and the glycan (the structure is shown in Figure 4A) precludes the detection of significant paramagnetic effects on the glycan signals of this molecule and therefore the paramagnetic approach was not used in this work. No STD signals were detected for the tag protons. Therefore, there is no interaction of the tag with the protein and the chelating unit does not interfere with the glycan interaction.

#### NA enzyme kinetics:

Total or absolute N2 enzyme activity was measured against the commonly used fluorescent substrate 2’-(4-Methylumbelliferyl)-α-D-N-acetylneuraminic acid (MUNANA; Sigma). MUNANA stocks were prepared fresh before each experiment and diluted to 100 μM (final) in 1x PBS, pH7.0, containing 1 mM CaCl_2_, 1 mM MgCl_2_. All reactions were incubated at 37 °C. At 2, 5, 10, 20-minute time points, 10 μL of the reaction mixture was sampled and diluted in 90μL of quenching solution consisting of 150 mM sodium carbonate, 150 mM glycine, pH 10, to stop the reaction. Fluorescence values were read on a BioTek Synergy microplate reader (excitation 365 nm; emission 445 nm) and activity in the form of leaving group released determined against a standard curve of 4-Methylumbelliferone.

Linkage specificity of recombinant NAs was measured using 4-nitrophenyl-α-N-Acetylneuraminic acid-(2–3)-β-D-galactopyranoside (NeuAc-α2–3-GalpNP) and 4-nitrophenyl-α-N-Acetylneuraminic acid-(2–6)-β-D-galactopyranoside (NeuAc-α2–6-GalpNP) as α2–3- and α2–6-linked substrates, respectively. Substrate stocks were diluted to 500 μM (final) in a reaction buffer consisting of 1x PBS, pH 7.0, containing 1 mM CaCl_2_, 1 mM MgCl_2_, and 20 U of β-galactosidase (Sigma). Reactions were incubated at 37°C, and colorimetric change (from leaving group release) read at 10, 20, 30, 40, 50, 60 minute time points BioTek Synergy microplate reader (absorbance 450 nm). All reactions were performed in triplicate and the data were analyzed using GraphPad Prism.

### QUANTIFICATION AND STATISTICAL ANALYSIS

Fluorescence intensities recorded using Mapix (Innopsys) for glycan microarray experiments in Figures 1 – 3, 6, 7, S1, S6, & S7 were quantified via measurement of mean intensity minus mean background of the four median out of six total replicate spots. Data presented are the average of these four replicates with standard error. Statistical analysis was not applied.

X-ray crystallographic data collection, solution, and refinement statistics are presented in Supplementary Data S4

## Supplementary Material

Supplemental **Supplementary Data Table S1:** Glycan array compound list. Related to STAR Methods, Figures 1 – 3, 6, 7, S1, S6, & S7.
**Supplementary Data Table S2:** Complete list of all glycan microarray results shown in full native bar graph form (as opposed to heat map or a condensed form). Related to STAR Methods, Figures 1 – 3, 6, 7, S1, S6, & S7.
**Supplementary Data S3:** Multiple sequence alignment of all studied H3 HAs leading up to (and within) the emergence of clades 3C.**2a** and 3C.**3a**. Related to Figures 2, 3, 5, 6, S4, & S5.
**Supplementary Data Table S4:** H3 HA receptor analog X-Ray data refinement and validation statistics. Related to STAR Methods, Figures 5, S3, & S5.
**Supplementary Data Table S5:** Supplementary glycan microarray document based on MIRAGE guidelines. Related to STAR Methods, Figures 1 – 3, 6, 7, S1, S6, & S7.
**Supplementary Data Table S6:** Supplementary glycan array MIRAGE data table. Related to STAR Methods, Figures 1 – 3, 6, 7, S1, S6, & S7.
**Supplementary Data Table S7:** Amino acid sequences of all recombinant H3 HA (rHA) ectodomains used within this study. Related to STAR Methods.

## Data Availability

Data for all novel X-ray crystal receptor complex structures have been uploaded to the Protein Data Bank (PDB) with accession codes 8TJ6 for Bei/89 H3 HA in complex with 6’-SLN, 8TJ4, 8TJ7, 8TJ8, 8TJ9, 8TJA and 8TJB for Bgk/79 H3, Sh/93 H3, Mos/99 H3, Mich/14 H3, Ecu/16 H3 and Tex/17 H3 HAs in complex with 6’-SLNLN, respectively. All datasets for glycan microarray experiments are attached in Supplementary Tables and Data files S5 & S6 according to the MIRAGE consortium format. These datasets have not been uploaded to a public repository since, at present, such a resource does not exist.
