# Supplementary material for: Evolution of human H3N2 influenza virus receptor specificity has substantially expanded the receptor-binding domain site
Source: Cell Host Microbe. Author manuscript; Available in PMC 2024 Apr 29. (PMC11057904; doi:10.1016/j.chom.2024.01.003)
Supplement: Supplemental — Supplementary Data Table S1: Glycan array compound list. Related to STAR Methods, Figures 1 – 3, 6, 7, S1, S6, & S7. Supplementary Data Table S2: Complete list of all glycan microarray results shown in full native bar graph form (as opposed to heat map or a condensed form). Related to STAR Methods, Figures 1 – 3, 6, 7, S1, S6, & S7. Supplementary Data S3: Multiple sequence alignment of all studied H3 HAs leading up to (and within) the emergence of clades 3C.2a and 3C.3a. Related to Figures 2, 3, 5, 6, S4, & S5. Supplementary Data Table S4: H3 HA receptor analog X-Ray data refinement and validation statistics. Related to STAR Methods, Figures 5, S3, & S5. Supplementary Data Table S5: Supplementary glycan microarray document based on MIRAGE guidelines. Related to STAR Methods, Figures 1 – 3, 6, 7, S1, S6, & S7. Supplementary Data Table S6: Supplementary glycan array MIRAGE data table. Related to STAR Methods, Figures 1 – 3, 6, 7, S1, S6, & S7. Supplementary Data Table S7: Amino acid sequences of all recombinant H3 HA (rHA) ectodomains used within this study. Related to STAR Methods. [file NIHMS1985284-supplement-Supplemental.pdf]

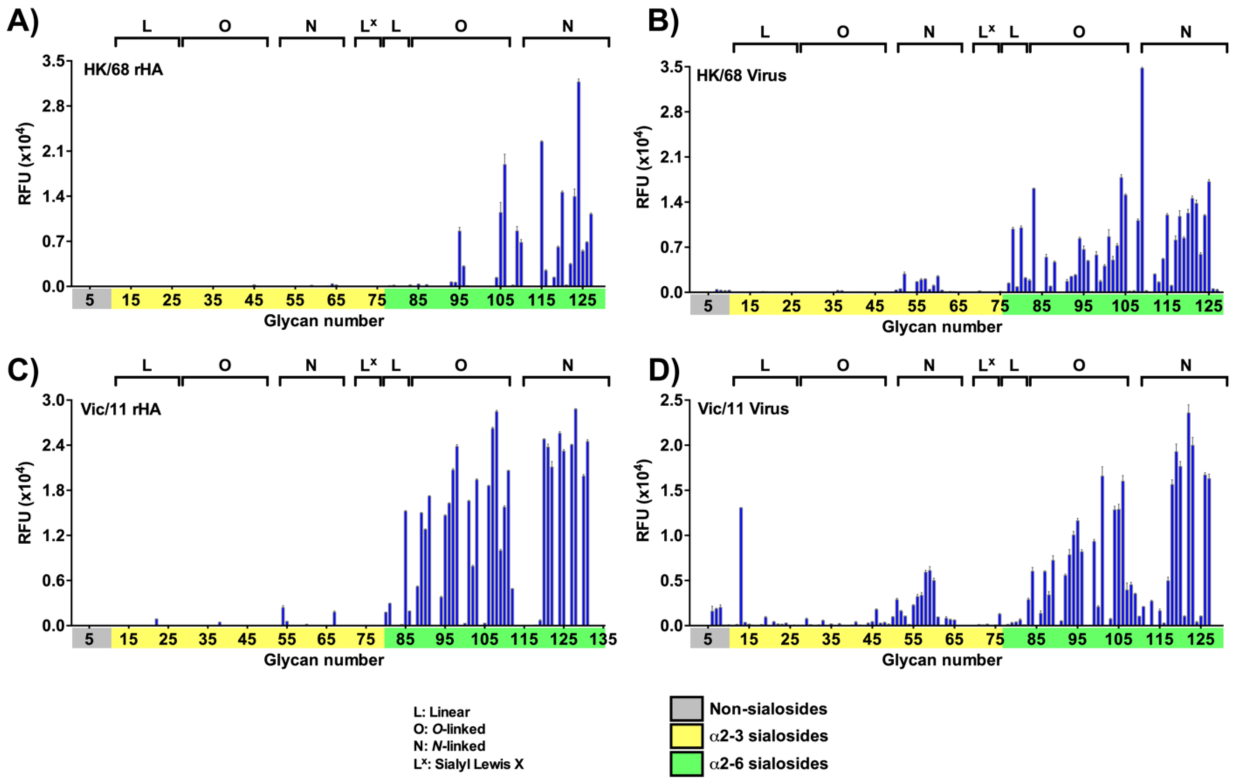

Figure S1. Modern human H3N2 IAVs restrict binding to elongated sialoside receptors.

Glycan array data confirming prior findings in Peng, de Vries *et al.*[15], that hemagglutinins from recent human H3N2 influenza viruses exhibit narrowed receptor specificity for glycans with extended (n = 3+) LacNAc disaccharide repeats beneath the terminal sialoside sugar. Panels A through D show example strains A/Hong Kong/01/68 (HK/68; A & B) compared to A/Victoria/361/2011 (Vic/11; C & D) as recombinantly-expressed H3 proteins ("rHA"; A & C) vs whole H3N2 virus samples ("Virus"; B & D). Length selectivity can be observed as absent/low intensity bars in the 2011 samples, particularly within the N-linked glycan regions. While comparison of panels A & C (rHAs) appears to show gains in binding to O-glycans and linear structures, these differences are likely due differences in stability between the early and late protein samples. Whole-virus samples in panels C & D show a far closer match in overall glycan specificity, with the exception of the evolved length-selectivity that is visible as absent/low-intensity bars through particularly the N-glycan region (note glycans 123, 126, & 129 in panel C and glycans 121, 124 & 125 in panel D). Panels A – D represent Sialoside array versions V3, V5, V1, & V3, respectively. Receptor structures corresponding to glycan numbers for all array versions can be found in Supplementary Data S1. Whole-virus staining was conducted using *Galanthus nivalis* lectin (GNL; see Star Methods and details in Thompson *et al.*[41]). n.b. apparent low-affinity binding to α2-3 linked receptors and some weak binding to short α2-6 receptors within Vic/11 virus sample is likely due to weak background NA-mediated binding as has been previously reported in the literature.

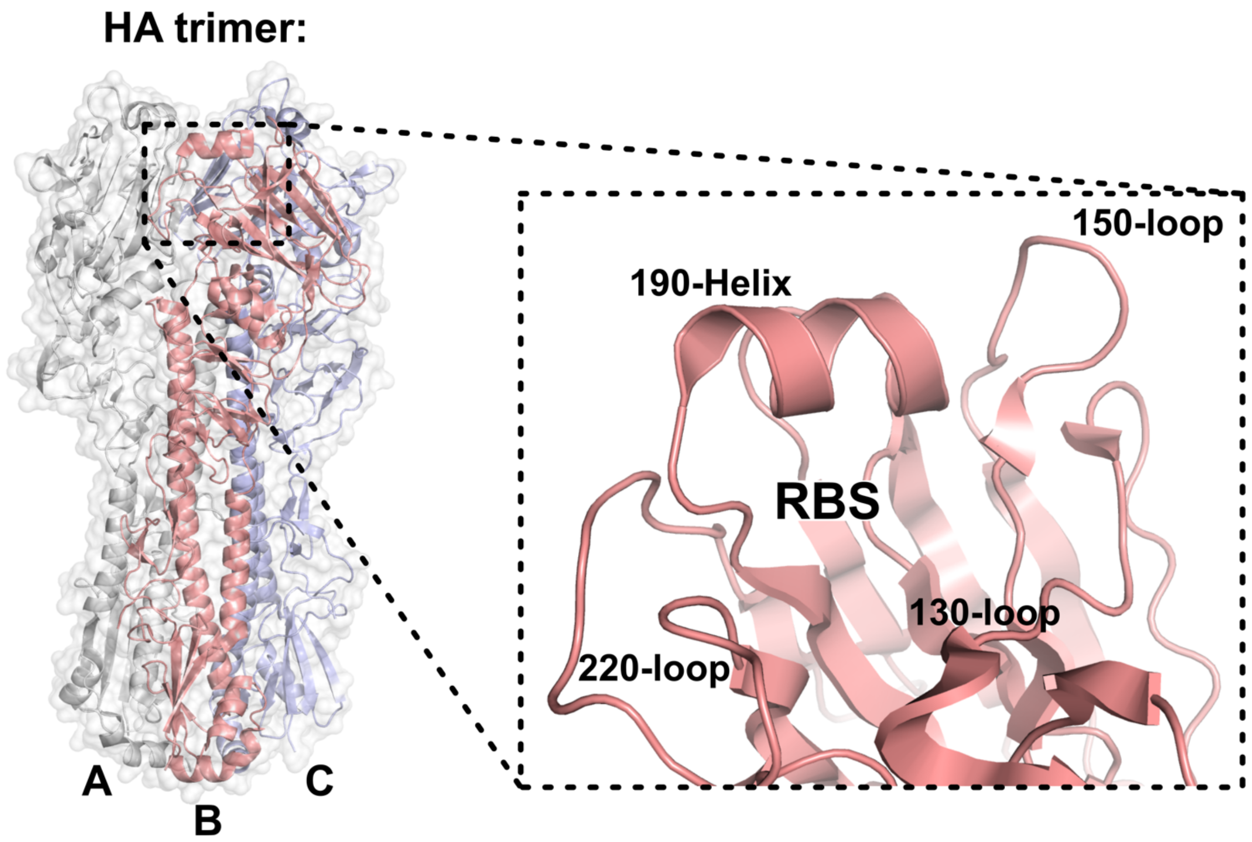

Figure S2. Structural overview of influenza HA

The HA glycoprotein of influenza (H3N2) viruses is a two-domain homotrimer, represented here as molecules A, B, & C shown in silver, pink, and blue, respectively; featuring HA head and stem domains. The canonical receptor binding site (RBS; shown highlighted via a black dashed box) is located in the upper part of the head domain and (shown inset) is made up of three well-characterized structural motifs, the 130/140 loop region, the 190 helix, and 220 loop. The 150 loop, located to the side of the RBS, is traditionally associated with antigenicity and a target for antibody binding. However, highly conserved Trp-153 at the base of the 150 loop is a key residue for sialic acid receptor binding. The example HA structure shown here is of A/Hong Kong/01/68 H3N2 (HK/68; PDB ID 6TZB). Figure assembled using PyMOL (Schrodinger, LLC).

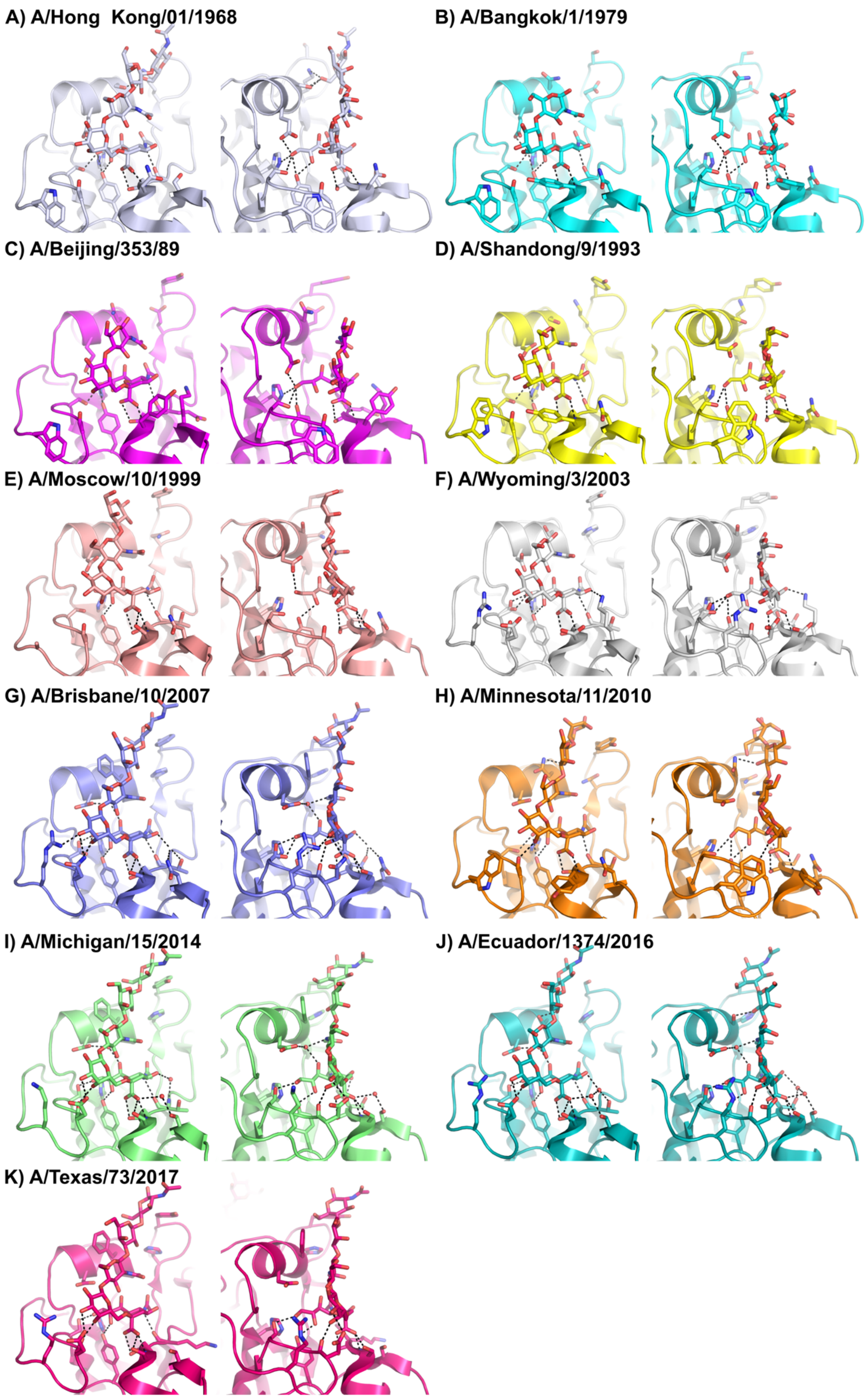
A) A/Hong Kong/01/1968
B) A/Bangkok/1/1979
C) A/Beijing/353/89
D) A/Shandong/9/1993
E) A/Moscow/10/1999
F) A/Wyoming/3/2003
G) A/Brisbane/10/2007
H) A/Minnesota/11/2010
I) A/Michigan/15/2014
J) A/Ecuador/1374/2016
K) A/Texas/73/2017

Figure S3. H3-receptor complex structures

Panels A – K show candidate receptor terminal fragments, either α2-6-sialyl-LacNAc (6SLN; panels C and F), LSTc (panel H), or α2-6-sialyl-$(LacNAc)_2$ ($6SLN_2$; all other panels), bound to representative H3 influenza glycoproteins covering approximately 3 – 10 yearly intervals over the last five decades. All panels represent identical views of the respective H3 complexes and feature two images of the RBS shown at 90° rotations around the y-axis relative to one another. Key receptor-binding side chains of residues 98, 135 – 137, 145, 156, 159, 183, 190, 193, 222, 225, & 225, are shown in all panels (see enlarged versions of panels A & K in Figure 5 for labels); key highly-coordinate water molecules indicating indirect receptor-binding interactions are shown as red spheres, while H-bonds are depicted as black dashed lines. Panels A, F, G, & H show previously published structures (PDB IDs 6TZB, 6BKR, 6AOV, & 5XRS, respectively), while other panels represent novel structures as part of this work. Panels assembled using PyMOL (Schrodinger, LLC).

A) NextStrain H3 clade distribution

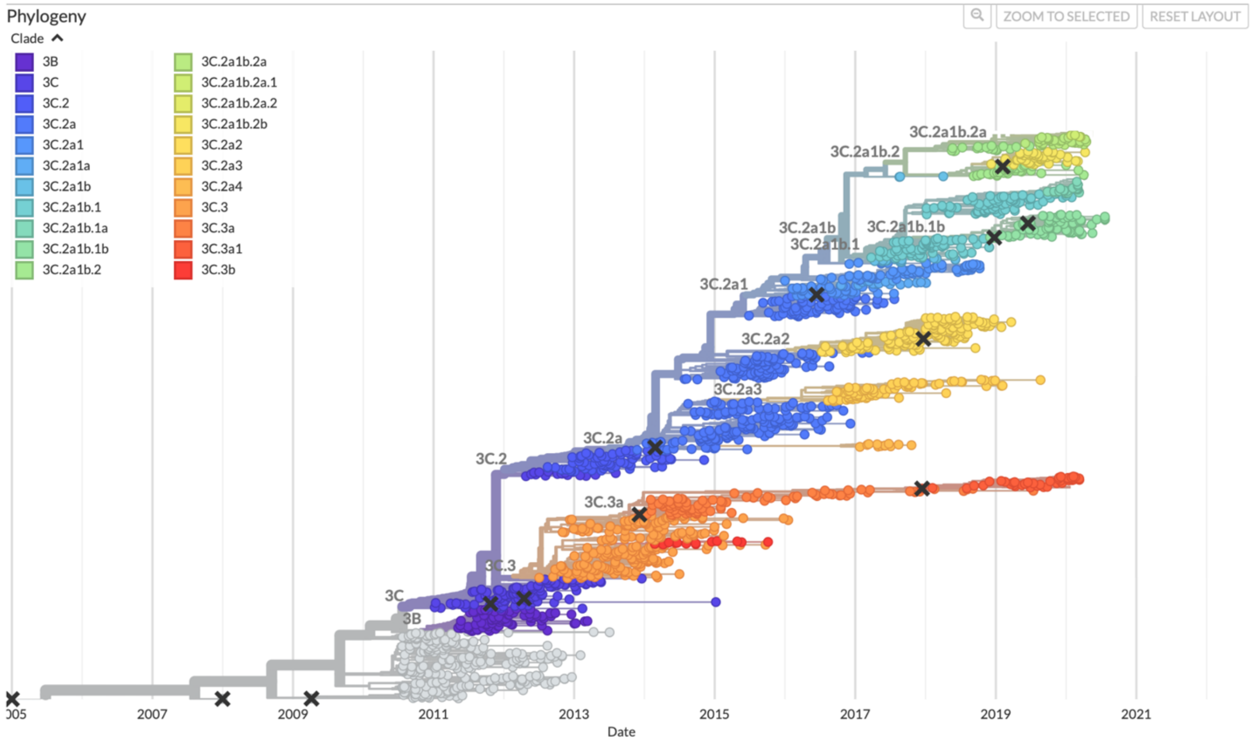

B) NextStrain H3 residue 159 identity by clade

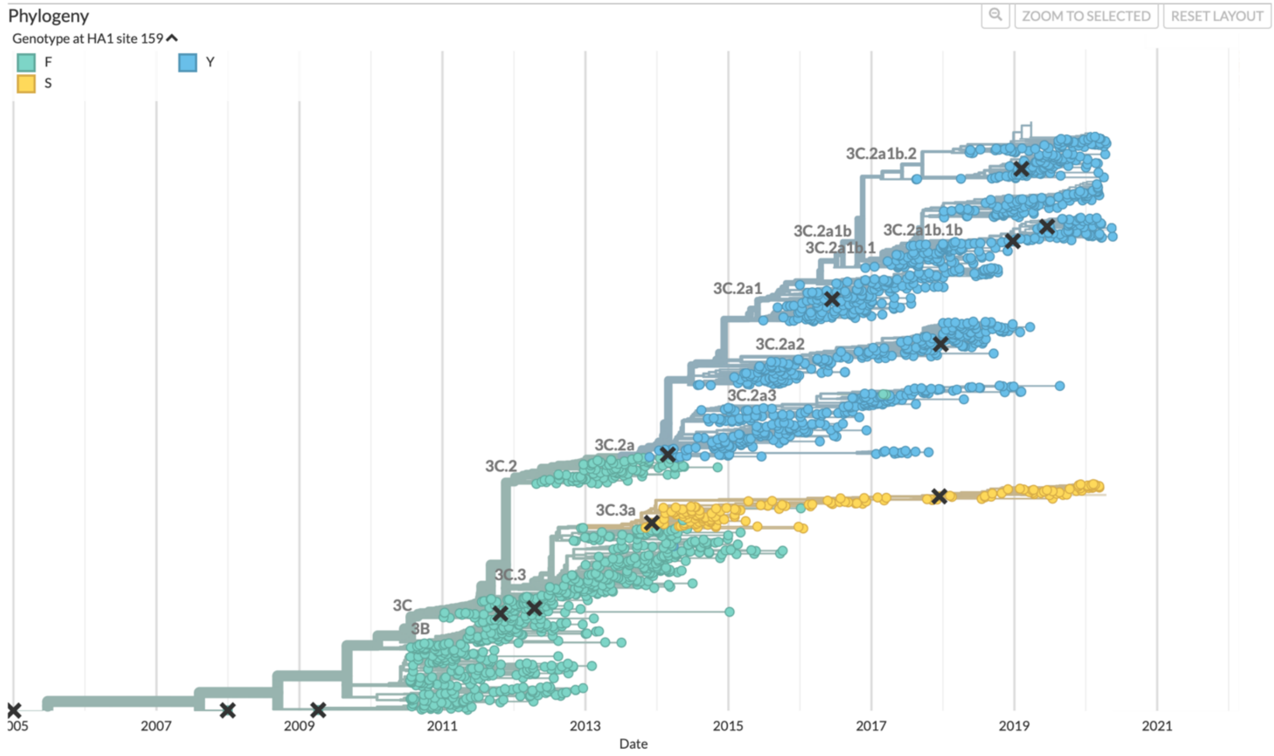

Figure S4. Nextstrain influenza clade phylogeny

Phylogeny trees of human H3N2 viruses over the last 1.5 decades produced by Nextstrain (https://nextstrain.org) and colored by (A) clade and (B) residue identity at HA position 159. As shown,

following evolution from F159 around 2013 onwards, Y159 or S159 diverge and are found exclusively in either clades 3C.2a-descended or 3C.3a-descended strains, respectively. n.b. Bold “X” markings depict vaccine strains selected within the different clades over time.

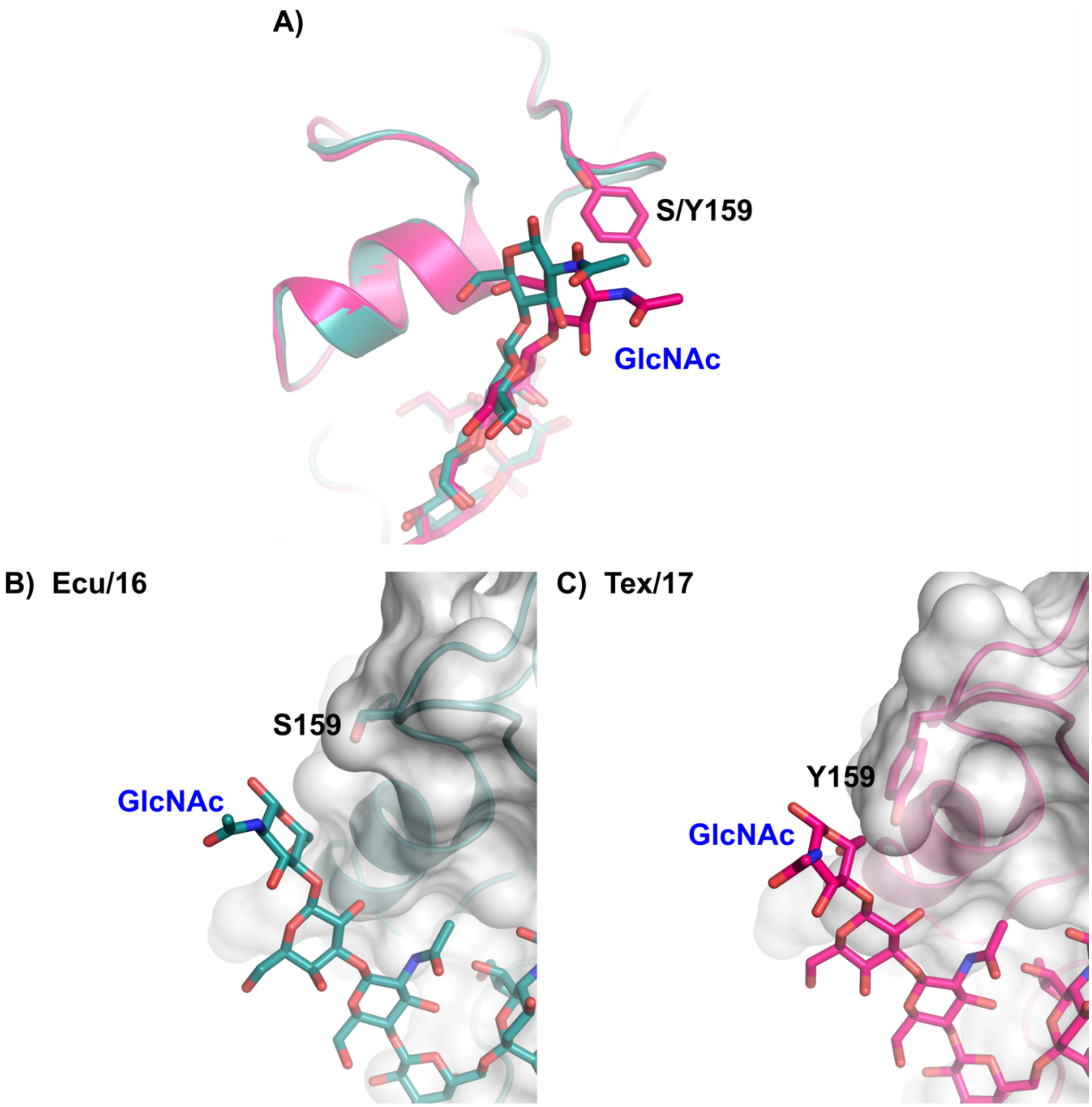

Figure S5. Structural comparison of receptor binding in clade 3C.2a and 3C.3a H3 HAs

Close up structural views of the 150-loop in 6SLN$_2$-receptor bound H3 representatives from clades 3C.2a (A/Texas/73/2017, Tex17; pink) and 3C.3a (A/Ecuador/1374/2016, Ecu16; teal). (A) Reduced steric interference from the smaller S159 side chain in 3C.3a HAs allows greater freedom at the reducing end of the receptor molecule, allowing the terminal GlcNAc sugar to rotate freely compared to the relatively fixed position in 3C.2a and earlier H3 structures. (B) & (C) Surface comparisons showing the substantial additional steric bulk imposed by Y/F159 side chains, compared to S159. Larger aryl-containing side chains push the reducing-end GlcNAc out from the HA surface, while substantial interactions with sugars below this position hold the glycan chain close to the surface. Thus longer saccharide chains with potentially greater flexibility are required to navigate this added surface complexity. Panels assembled using Pymol (Schrodinger, LLC).

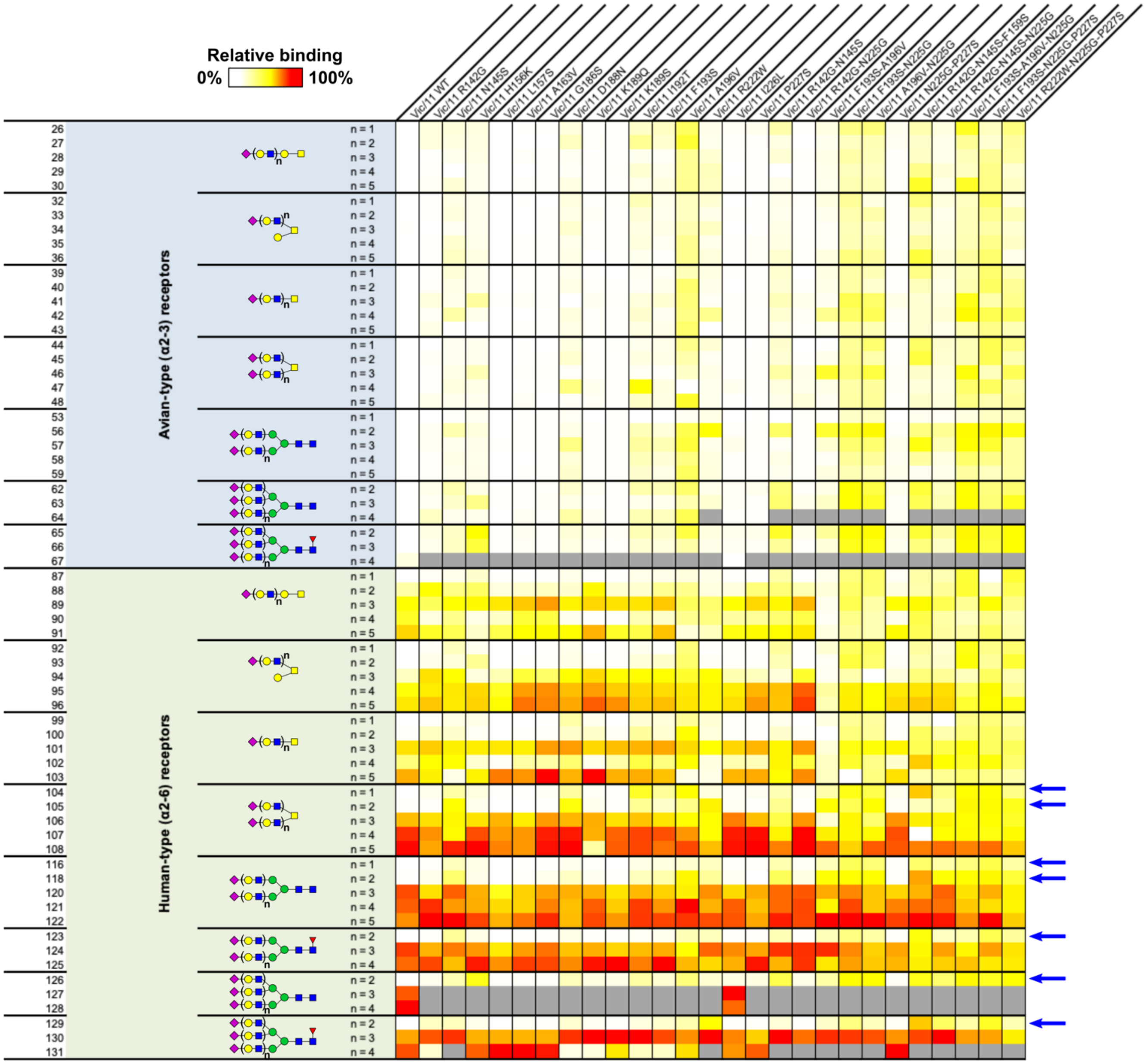

Figure S6. Glycan microarray analysis of Vic/11 to HK/68 RBS reversion mutants

Heatmap representation of glycan microarray data comparing Vic/11 WT to RBS-proximal variants where amino acid species have been reversed to their original identity in the post-pandemic HK/68 strain. Only variants giving rise to detectable binding are shown. WT Vic/11 (far left column) maintains a characteristic pattern of strong binding to extended receptors with little or no interactions with shorter or α2-3-terminal glycans. All variants, as far as a 13-mutant HA, result in either no binding (not shown in this figure) or limited alteration of the WT Vic/11 specificity pattern (compare columns moving left to right). The final triple mutants shown on the right of the panel reveal the beginnings of a non-binding phenotype, with native specificity almost entirely lost and widespread non-specific binding to almost all receptors visible.

The color scale within individual columns (array datasets) are independently scaled to the most intense RFU within that group. Blue arrows highlight short receptors within a given group containing only 2 LacNAc repeats. Columns depict data collected from various different Sialoside array versions, including V1, V3, V4, & V5. Receptor structures corresponding to glycan numbers for all array versions can be

found in Supplementary Data S1. Grey bars illustrate that a given receptor structure was absent in the array version on which a particular dataset was collected. Individual array plots for all mutants, including binders shown here and non-binders, are illustrated in Supplementary Data S2.

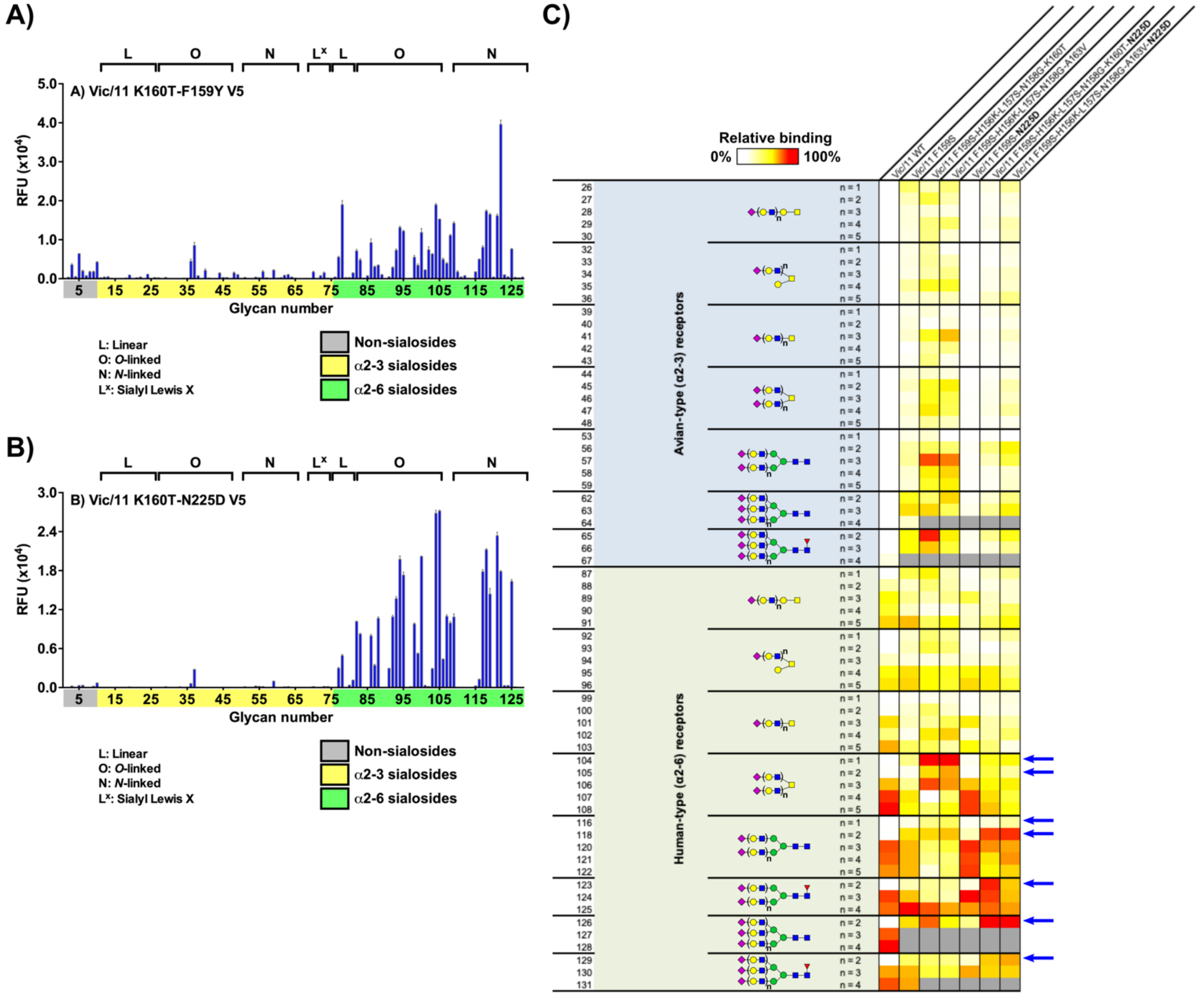

Figure S7. Glycan microarray analysis of variants restoring receptor binding in mutated Vic/11 backgrounds

Addition of two variants, F159Y (A) and N225D (B), that evolved immediately before K160T in clade 3C.2a H3s restore receptor binding in a Vic/11-K160T background that alone inhibits receptor engagement through creation of a novel glycan at position N158. While both F159Y and N225D promote positive binding compared to K160T alone, the K160T-N225D double variant in panel (B) clearly shows the most native-like receptor specificity, indicating this variant has the dominant positive effect. Panel (C) shows a glycan microarray heatmap of WT Vic/11 compared to RBS-proximal variants reverted to respective HK/68 amino acid species without and with N225D. Latter versions (righthand 3 columns) all show stronger and far more comparable binding specificity to WT, while variants without N225D show substantially disrupted, non-specific binding.

The color scale within individual columns (array datasets) are independently scaled to the most intense RFU within that group. Blue arrows highlight short receptors within a given group containing only 2 LacNAc repeats. Columns depict data collected from various different Sialoside array versions, including V3, V4, & V5. Receptor structures corresponding to glycan numbers for all array versions can be found in Supplementary Data S1. Grey bars illustrate that a given receptor structure was absent in the array

version on which a particular dataset was collected. Individual array plots for all mutants are illustrated in Supplementary Data S2.
